# A hemolytic-uremic syndrome-associated strain O113:H21 Shiga toxin-producing *Escherichia coli* specifically expresses a transcriptional module containing *dicA* and is related to gene network dysregulation in Caco-2 cells

**DOI:** 10.1371/journal.pone.0189613

**Published:** 2017-12-18

**Authors:** Silvia Yumi Bando, Priscila Iamashita, Beatriz E. Guth, Luis F. dos Santos, André Fujita, Cecilia M. Abe, Leandro R. Ferreira, Carlos Alberto Moreira-Filho

**Affiliations:** 1 Department of Pediatrics, Faculdade de Medicina da Universidade de São Paulo (FMUSP), São Paulo, SP, Brazil; 2 Departament of Microbiology, Immunology and Parasitology, Universidade Federal de São Paulo, Escola Paulista de Medicina, São Paulo, SP, Brazil; 3 Department of Computer Science, Instituto de Matemática e Estatística, Universidade de São Paulo, São Paulo, SP, Brazil; 4 Laboratory of Bacteriology, Butantan Institute, São Paulo, SP, Brazil; University of Padova, Medical School, ITALY

## Abstract

Shiga toxin-producing (Stx) *Escherichia coli* (STEC) O113:H21 strains are associated with human diarrhea and some of these strains may cause hemolytic uremic syndrome (HUS). The molecular mechanism underlying this capacity and the differential host cell response to HUS-causing strains are not yet completely understood. In Brazil O113:H21 strains are commonly found in cattle but, so far, were not isolated from HUS patients. Here we conducted comparative gene co-expression network (GCN) analyses of two O113:H21 STEC strains: EH41, reference strain, isolated from HUS patient in Australia, and Ec472/01, isolated from cattle feces in Brazil. These strains were cultured in fresh or in Caco-2 cell conditioned media. GCN analyses were also accomplished for cultured Caco-2 cells exposed to EH41 or Ec472/01. Differential transcriptome profiles for EH41 and Ec472/01 were not significantly changed by exposure to fresh or Caco-2 conditioned media. Conversely, global gene expression comparison of both strains cultured in conditioned medium revealed a gene set exclusively expressed in EH41, which includes the *dicA* putative virulence factor regulator. Network analysis showed that this set of genes constitutes an EH41 specific transcriptional module. PCR analysis in Ec472/01 and in other 10 Brazilian cattle-isolated STEC strains revealed absence of *dicA* in all these strains. The GCNs of Caco-2 cells exposed to EH41 or to Ec472/01 presented a major transcriptional module containing many hubs related to inflammatory response that was not found in the GCN of control cells. Moreover, EH41 seems to cause gene network dysregulation in Caco-2 as evidenced by the large number of genes with high positive and negative covariance interactions. EH41 grows slowly than Ec472/01 when cultured in Caco-2 conditioned medium and fitness-related genes are hypoexpressed in that strain. Therefore, EH41 virulence may be derived from its capacity for dysregulating enterocyte genome functioning and its enhanced enteric survival due to slow growth.

## Introduction

Hemolytic uremic syndrome (HUS) is a thrombotic microangiopathy that is clinically defined by thrombocytopenia, non-immune hemolytic anemia, and acute renal failure. Typical HUS develops secondary to gastrointestinal infection with Shiga toxin (Stx)-producing *Escherichia coli* (STEC) [1]. The pathogenesis of STEC in intestinal illness usually entails attachment to the intestinal epithelial cells, followed by the secretion of Stx. Most STEC attach via the intimin adherence protein, encoded by the *eae* gene that resides on the locus of enterocyte effacement (LEE) pathogenicity island. There are, however, LEE-negative STEC strains, such as O113:H21, that do not produce intimin but can also cause HUS [2–5]. In fact, this serotype harbors several virulence genes, such as *sab*, *subAB*, *ehxA* and possibly other yet unknown virulence factors, but it is not clear how all these genes/factors act together with Stx in the infection pathogenesis [6].

Interestingly, in Brazil O113:H21 strains are commonly found in cattle but, so far, were not isolated from HUS patients [7, 8]. Brazil is the second beef cattle producer in the world and, with India, the largest exporter in 2016 [9, 10]. Therefore, it is important for food safety and public health to identify and characterize the O113:H21 strains isolated from cattle due to their virulence potential as human pathogens.

Genotypic and phenotypic studies have been performed in O113:H21 STEC strains isolated from food, environment, animal reservoir, and human infections aiming at characterizing the pathogens and the environmental strains [6, 7, 11, 12]. These studies also searched for molecular markers, such as virulence genes, for: i) discrimination of environmental strains from those associated with human diseases; ii) evaluation of the capacity of animal isolates in causing human diseases. However, HUS-associated and environmental-isolated STEC strains did not present clear differences, so the virulence potential of carcass-associated strains remained indeterminate.

Hence the molecular mechanism underlying the capacity to cause HUS and the differential host cell response to HUS-causing strains are not yet completely understood. In the present work we conducted gene co-expression network (GCN) in two O113:H21 STEC strains: i) one isolated from a HUS patient (EH41 reference strain); ii) the other isolated from bovine feces (Ec472/01). We also characterized the differential Caco-2 cells response after EH41 or Ec472/01 interaction. GCNs were based on differentially expressed genes and constructed using Pearson’s correlation in STEC strains or enterocyte cells. In STEC networks this approach permitted: i) the analysis of GCNs for differentially expressed genes; ii) the identification of transcriptional modules; iii) the identification of candidate genes as molecular markers able to distinguish between HUS-associated O113:H21 STEC strains and strains isolated from animal or environmental sources. In Caco-2 networks the GCN analysis characterized the differential enterocyte response for EH41 or Ec472/01 interaction.

## Materials and methods

**Fig 1:**
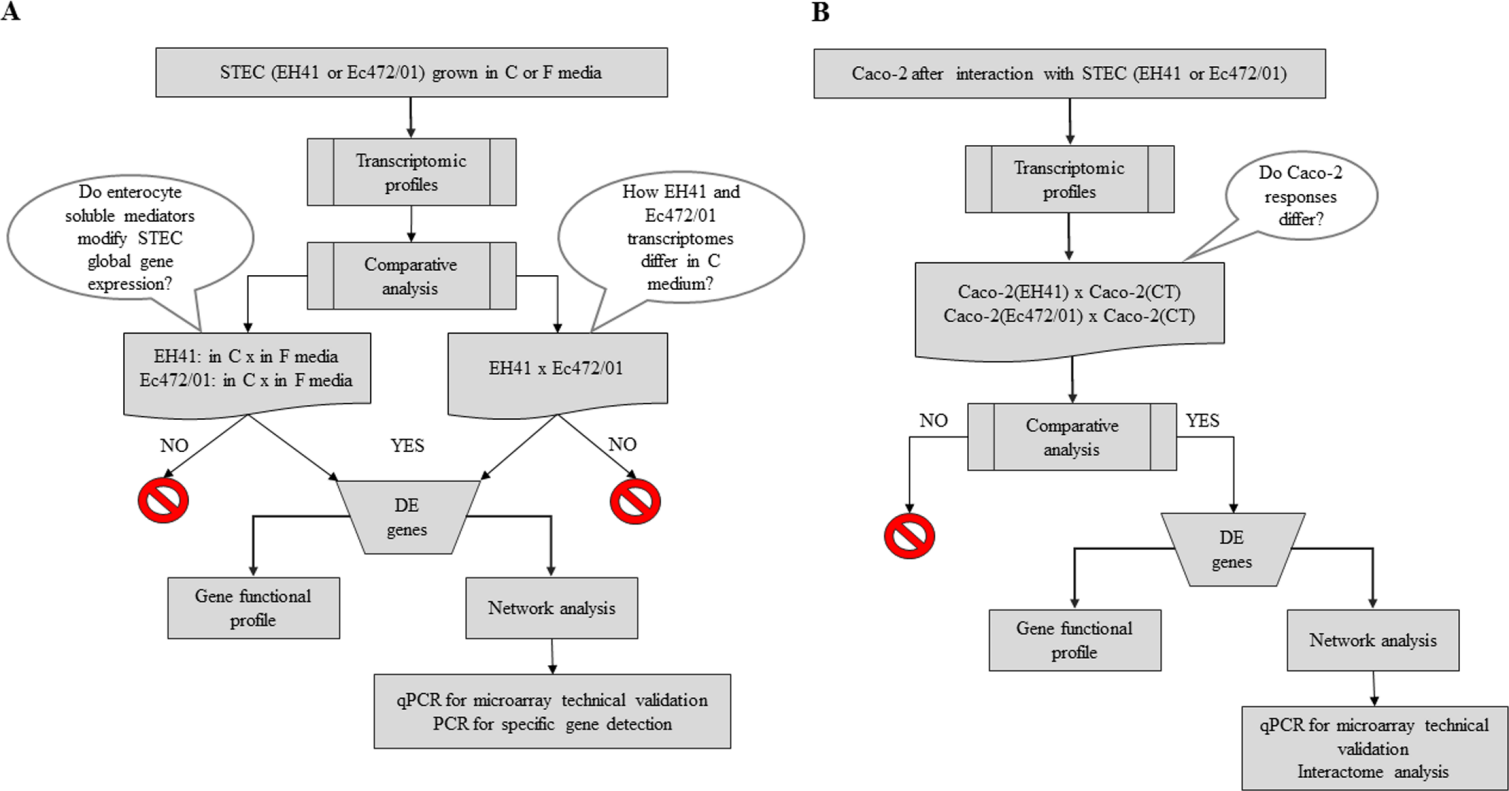
Workflow of gene co-expression network analyses for STEC strains and Caco-2 cells. (A) Network analysis for STEC strains. Two comparative analyses were done to investigate if enterocyte soluble mediators modify global gene expression after bacterial growth in Caco-2-conditioned (C) medium for 3h: i) EH41 in C medium X EH41 in F medium; ii) Ec472/01 in C medium X Ec472/01 in F medium. Another comparative analysis was done to assess global gene expression differences between EH41 and Ec472/01 in C medium. (B) Network analyses for Caco-2 cells after 3h of interaction with EH41 or Ec472/01. Two comparative analyses were done: Caco-2 exposed to EH41 X Caco-2 control and Caco-2 exposed to Ec472/01 X Caco-2 control.

Fig 1 shows the workflow adopted for the transcriptional analyses (comparative global gene expression and gene co-expression network analysis) of the two O113:H21 STEC strains: EH41, isolated from a HUS patient, and Ec472/01, isolated from cattle feces, both cultured in fresh or in Caco-2 cell line (enterocyte) conditioned media. Two additional comparative gene network analyses were performed for cultured Caco-2 cells exposed to EH41 or Ec472/01 bacteria.

### Bacterial strains

We used a total of 12 STEC strains of serotype O113:H21 (S1 Table) kept at Department of Microbiology, Immunology and Parasitology, Escola Paulista de Medicina, UNIFESP, Sao Paulo, Brazil. The serotype, cytotoxic activity and enterohaemolytic phenotype of all these strains were previously confirmed [7]. Global gene expression and gene network analyses were performed for two of these strains: EH41, isolated from a child with HUS in Australia [13], and Ec472/01 isolated from cattle feces in Brazil [7]. For all biological and molecular assays described in the next sections, a single colony of each strain was inoculated in trypticase soy broth and grown at 37°C for 18 h.

### Growth of strains EH41 and Ec472/01 in fresh (F) or conditioned (C) medium and RNA extraction

Fresh (F) medium stands here for antibiotic-free DMEM medium containing 10% fetal bovine serum (FBS). The same medium recovered after 24 h of incubation with Caco-2 cells is designated conditioned (C) medium. F or C media were subsequently used for bacterial growth assays involving the strains EH41 or Ec472/01. Briefly, 400 μL of bacterial culture (Abs_550_ = 0.35) were inoculated in 4 mL of F or C medium and incubated for 3 h at 37°C. There were four biological replicates for each bacterial strain. After this period the bacteria were recovered by centrifugation for 10 min at 5000 xg and the pellet was suspended in 600 μL of RNAprotect Bacteria Reagent (Qiagen cat. no. 76506, Valencia, CA) for RNA preservation. Bacterial cells were lysed using lysozyme (1 mg/mL) and proteinase K (2 mg/mL) and incubated for 10 min at 20°C. Total RNA was obtained using the RNeasy Mini Kit (Qiagen cat no. 74104, Valencia, CA). RNA purity analysis and quantification was accomplished by using the NanoVue spectrophotometer. RNA integrity was assessed on the Agilent BioAnalyzer 2100 (Agilent, Santa Clara, CA). All samples presented RIN > 7.5 and were stored at -80°C until use in hybridization experiments.

### Enterocyte-STEC interaction assays

The Caco-2 cells were cultured in T flasks (25 cm^2^) or in 4 wells cell culture plates containing glass coverslips (13 mm) and DMEM with FBS (10%) and penicillin-streptomycin (100 U/mL-100 μg/mL) in a 5% CO_2_ at 37°C. The cells were grown until confluence and the formation of a polarized epithelial cell monolayer (it occurs between 5–7 days). Twenty-four hours prior to interaction assays the cells were washed three times with 1X phosphate buffered saline and incubated with 4 mL of antibiotic-free DMEM containing 10% fetal bovine serum. Interaction assays were performed with 400 μL or with 50 μL (Abs_550_ = 0.35) of bacterial culture respectively placed on a Caco-2 monolayer cultured in T flasks or in 4 wells cell culture plates. Subsequently, the cell cultures were incubated in 5% CO_2_ at 37°C for 3 hr. After this period, the Caco-2 cells that were exposed to bacteria and the uninfected controls—both in quadruplicates—were recovered for RNA extraction or for electron microscopy.

### RNA extraction

After enterocyte-STEC interaction, the cells were gently washed 3 times with 1X phosphate buffered saline. Subsequently, Caco-2 cells were recovered from the culture flasks by vigorous pipetting with 1X phosphate buffered saline. The cells were then centrifuged for 5 min at 5000 xg and the pellet was resuspended in 600 μL of RNAlater Reagent (Qiagen cat. no. 76154, Valencia, CA) for RNA preservation. Total RNA was extracted from Caco-2 cells after lysing with RLT buffer and the RNeasy Mini Kit (Qiagen cat no. 74104, Valencia, CA). RNA purity analysis and quantification was accomplished by using the NanoVue spectrophotometer. RNA quality was assessed on the Agilent BioAnalyzer 2100 (Agilent, Santa Clara, CA). All samples presenting RIN > 7.5 were stored at -80°C until use in hybridization experiments.

### Microarray hybridization

#### STEC strains

In order to determine the gene expression profiles for EH41 and EC472/01 strains, 15 K DNA microarrrays (*E*. *coli* Gene Expression Microarray, Agilent Technologies, cat no. G4813A-020097, Santa Clara, CA) were used. Cyanine 3-CTP fluorescent dye (Cy-3 dye) was used for hybridization labeling (Fairplay III Labeling, version april/2009, Stratagene, adapted for one-color protocol).

#### Caco-2 cells

Gene expression profiles for Caco-2 cells were obtained using 44 K DNA microarrrays (Whole Human Genome Microarray Kit, Agilent Technologies, cat no. G4112F, Santa Clara, CA) were used. Cy-3 dye was used for hybridization labelling (One-Color Microarray-Based Gene Expression Analysis—Quick Amp Labeling).

### Data acquisition and processing

The microarray images were captured by the reader Agilent Bundle according to the parameters recommended for bioarrays and extracted by Agilent Feature Extraction software version 9.5.3. Spots with two or more flags (low intensity, saturation, controls, etc.) were considered as NA, that is, without valid expression value. The R software version 2.11.1 and an in house script were used for: i) sample grouping (the comparison groups are described in Table 1); ii) excluding transcript spots presenting three or more NAs per group; iii) converting gene expression values to log base 2 [14,15]. Through this procedure the valid transcripts were obtained for each of the comparison groups (Table 1). TMEV software version 4.6.1 and Significance Analysis of Microarrays (SAM) was used for obtaining the differentially expressed genes/ transcripts for all comparisons. All array data is available from the GEO database (https://www.ncbi.nlm.nih.gov/geo/, accession number GSE45979).

**Table 1:** Differentially expressed (DE) and exclusively expressed (EE) valid transcripts obtained for each of the comparison groups.

		DE genes/transcripts		
Comparison group	Valid transcripts	hyperexpressed	hypoexpressed	EE genes
EH41in C medium vs EH41in F medium	3,802	3	4	31^a^
Ec472 in C medium vs Ec472 in F medium	3,802	42	3	8^a^
EH41in C medium vs Ec472 in C medium	5,823	15	82	41^b^, 35^c^
Caco2-EH41 vs Caco2-Control	21,116	79	12	NA
Caco2-Ec472 vs Caco2-control	20,960	127	9	NA

^a^EE transcripts in conditioned (C) medium

EE transcripts obtained for EH41^b^ or for Ec472/01^c^ in C medium.

### Gene co-expression networks (GCNs)

Gene co-expression networks (GCNs) were constructed by using Pearson’s correlation. Data analysis, hierarchical network structure, and visualization were accomplished through Cytoscape (version 3.1.0, www.cytoscape.org). All networks were tested for scale-free status, i.e. power law distributions in empirical data [15]. The GCN correlation thresholds were chosen in order to ensure that most of nodes continued to be connected to the major component and that the network remained stable along a threshold range, i.e., maintaining network’s topological structure [14].

### Venn diagram analysis

We used a web tool Draw Venn Diagram–UGent (available at http://bioinformatics.psb.ugent.be/webtools/Venn/) for analyzing DE gene data sets.

### PCR analysis for specific gene detection in a panel O113:H21 STEC strain

We used Primer-BLAST (Primer3 Input, version 0.4.0 and BLAST, available at http://www.ncbi.nlm.nih.gov/tools/primer-blast/) for designing specific primers (S2 Table) in order to search for selected genes in a panel of STEC O113:H21 strain encompassing the 12 STEC strains used in this study (S1 Table). Each PCR reaction was performed in a final volume of 25 μl containing 1.5 U of High-Fidelity Platinum Taq DNA Polymerase (Invitrogen, Carlsbad, CA, USA), 20 mM Tris-HCl (pH 8.4), 50 mM KCl, (1.0–2.0) mM MgSO4 (S2 Table), 150 μM each of dATP, dCTP, dGTP and dTTP, 0.3 μM of primers and 1 μl of a boiled bacterial suspension as the DNA template. The amplification conditions consisted in 30 cycles of 95°C for 30 s, (57–60)°C (S2 Table) for 30 s and 72°C for 1 min, with an initial denaturing step of 95°C for 5 min and a final extension step of 72°C for 5 min. After PCR, 5-μl aliquots of the amplification products were electrophoresed in 1.5% agarose gels in 1×TAE buffer (40 mM Tris, 20 mM acetic acid, 1 mM EDTA). The samples were stained with GelRed^TM^ Nucleic Acid Gel Stain (cat. 41003, Biotium, Hayward, CA) and DNA bands were visualized using UV light. The 1 Kb Plus DNA Ladder (cat. 10787–018, Invitrogen) was used as molecular size markers in all gels.

### qPCR for microarray technical validation

Microarray expression data for bacterial strains or Caco-2 cells were validated through quantitative real-time polymerase chain reaction (qPCR). Specific primers for all selected genes (S3 Table) were designed using the Primer-BLAST (Primer3 Input, version 0.4.0 and BLAST, available at http://www.ncbi.nlm.nih.gov/tools/primer-blast/). All samples were amplified in triplicate (technical replicates). Real time PCR amplifications were performed in Applied Biosystems StepOne Plus Real Time PCR System with StepOne software (Applied Biosystems, Forrest City, CA, USA). All RNA samples were previously treated with DNAse and checked for DNA contamination by PCR analysis.

### Validation of STEC microarrays

Amplification reactions were performed in a 20 μL final volume containing 1X RT Enzyme Mix and 1X RT-PCR Mix (Power SYBR Green RNA-to-C_T_ 1-Step, Applied Biosystems, Carlsbad, CA), 5 pmol of primers and 100 ng of total RNA. We used the following cycling parameters: a RT step of 48^o^ C for 30 min, an enzyme activation of 95°C for 1 min followed by 40 cycles of 95°C for 15 s and 60°C for 1 min. In order to normalize qPCR reactions, *rpo*A was included as reference gene. Relative gene expression was determined by the relative standard curve method and presented as relative expression using *rpo*A as endogenous control for STEC strains.

### Validation of Caco-2 microarrays

Amplification reactions were performed in a 25 μL final volume containing 1X SYBR Green mix (*Quantitec SYBR Green PCR kit*, QIAGEN, Hilden, DE), 10 pmol of primers and 2 μL cDNA (1/10 dilution, synthesized from 1μg of total RNA). We used the following cycling parameters: an initial hot start of 95°C for 15 min followed by 50 cycles of 95°C for 15 s and 60°C for 30s. In order to normalize qPCR reactions, *GAPDH* was included as reference gene. Relative gene expression was determined by the relative standard curve method and presented as relative expression using *GAPDH* as endogenous control for Caco-2 infected by STEC EH41 or by EC472 and for uninfected Caco-2.

### Bacterial growth assessment for EH41 and Ec472/01

The bacterial growth was evaluated by absorbance readings at 550 nm (Multiskan MS Primary EIA, Labsystems, MA) just before and after 3h of growth in C medium.

### Scanning electron microscopy (SEM) for Caco-2 cells

After Caco-2 cell-STEC interaction, the cells exposed to bacteria and the uninfected controls were gently washed 3 times with 1X phosphate buffered saline (PBS) and fixed with Karnovsky fixative solution for at least 24 h at 4°C. After fixation, cells were washed 3 times with 0.1 M cacodylate buffer (10 min) and post- fixed with 1% osmium tetroxide (prepared in the same buffer) for 30 min. After being washed for 3 times with distilled water, preparations were dehydrated through a graded ethanol series (50%, 75%, 85%, 95%, and 100%). Subsequently, the preparations were dried (critical point method), mounted on stubs and sputter coated with gold. Specimens were then examined under SEM (QUANTA 250—FEI Company, Netherlands) at 12.5 kV.

## Results

In this study (see Fig 1) we investigated the comparative global gene expression of two STEC strains–EH41, reference strain isolated from a patient with HUS, and Ec472/01, isolated from cattle feces–after bacterial growth in Caco-2 cells conditioned (C medium) or fresh media (F medium). Another comparative analysis was performed for Caco-2 cells after 3h of *in vitro* bacteria-host cell interaction. We obtained GCNs based on differentially expressed genes for Caco-2 cells and for both STEC strains, as described below.

### Transcriptional analysis of STEC EH41 and Ec472/01 growth in C and F media

**Fig 2:**
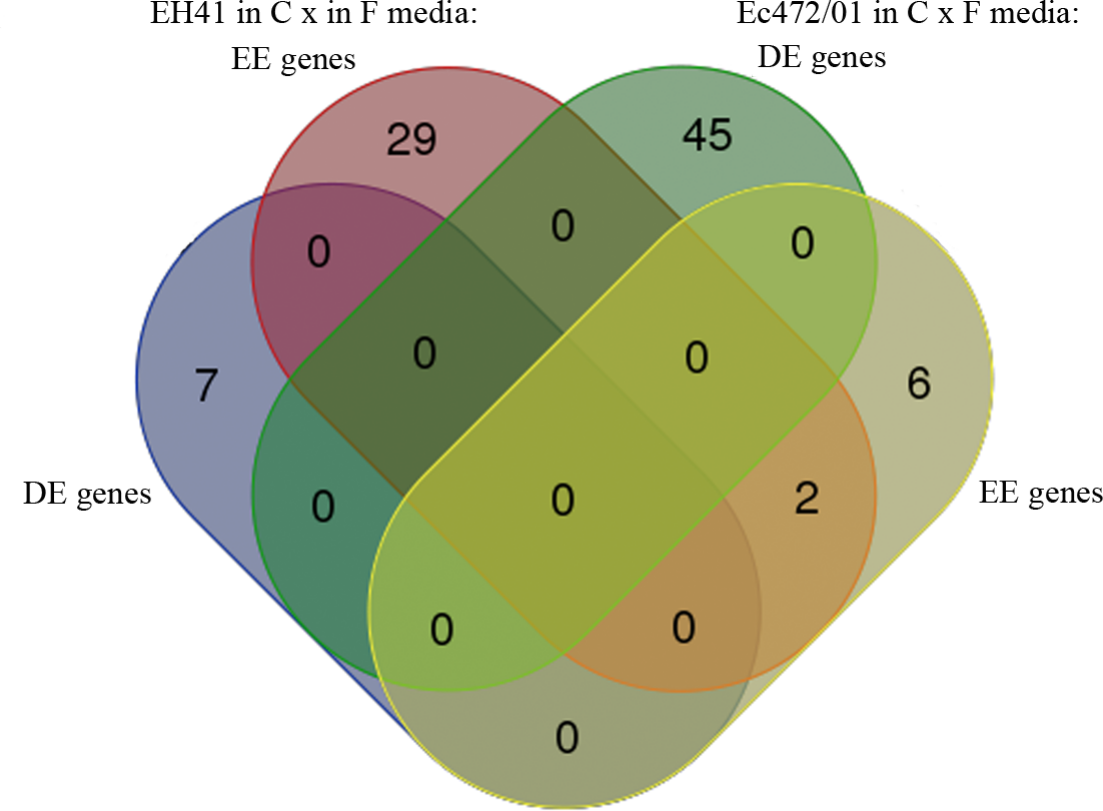
Differential gene expression profiles for STEC strains. Venn diagram analysis for DE and EE genes obtained from two comparisons: EH41 in C medium X EH41 in F medium or Ec472/01 in C medium X Ec472/01 in F medium.

Our initial analysis aimed at investigating if enterocyte soluble mediators modify STEC global gene expression. In order to accomplish this goal we performed a comparative analysis by growing the two STEC strains in C or F medium for 3 hours. Comparative gene expression analysis of STEC strains cultured in C or F medium revealed that 38 transcripts for EH41 and 53 transcripts for Ec472/01 are differentially expressed in C medium (Table 1). The biological functions of these genes are listed in S4 and S5 Tables. Venn diagram analysis of these differentially expressed transcripts (Fig 2) showed that only two transcripts (*ECs1070* and *ECs4328*, both functionally uncharacterized) are common between EH41 and Ec472/01 strains. Noteworthy, none of these transcripts is a known or putative virulence factor. On the other hand, it is well described that the expression of virulence factors is modulated by enterocyte mediators [16]. Therefore, our results indicate that HUS-associated STEC (EH41) and Ec472/01 strains have their own “pre-exposure” differential transcriptomic profile.

**Fig 3:**
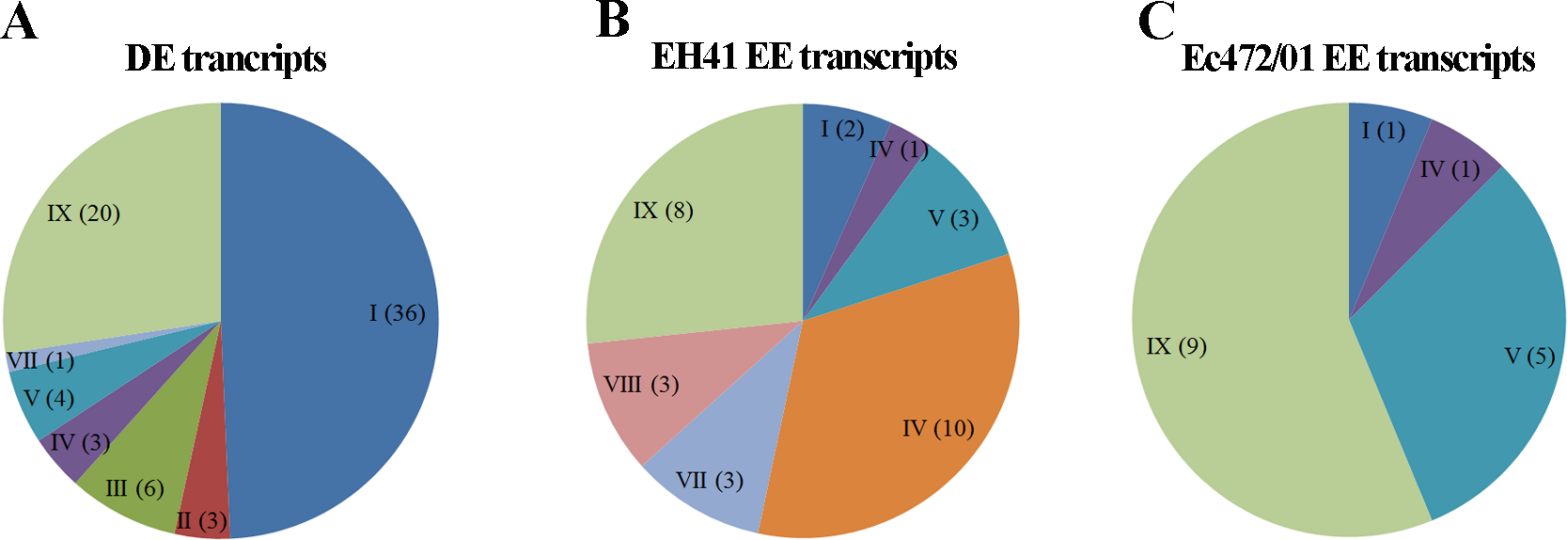
Functional profile analyses of DE and EE transcripts obtained from the comparison of EH41 X Ec472/01 in C medium. Pie charts show: (A) the DE transcripts set; (B) the EE transcripts in EH41; (C) the EE transcripts in Ec472/01. Functional categories are identified by roman numerals as follows: I, metabolic process; II, chaperone; III, fimbrin/ outer membrane protein; IV, ion transport/ protein transport; V, transcription; VI, qin prophage/ prophage; VII, acid resistance; VIII, transposase; IX, putative protein. The number of genes belonging to a particular functional category is indicated between parentheses in each slice. Transcripts described as hypothetical or unknown proteins are not represented in this figure.

Consequently, we conducted a comparison between EH41 and Ec472/01 exposed only to the C medium. All differentially expressed (DE) and exclusively expressed (EE) transcripts (Table 1) and their biological functions are listed in S6 Table. The functional profile analysis of the DE and EE transcripts, except for hypothetical or unknown protein, are shown in Fig 3. Almost half of the DE transcripts (49%) are involved in metabolic process (Fig 3A), whereas 33% of EE transcripts in EH41 codify prophage-derived genes and three genes are involved in acid resistance (Fig 3B). Only seven Ec472/01 EE transcripts have a known biological function (Fig 3C). Interestingly, the majority of DE transcripts are hypoexpressed in EH41 (S6 Table) and are involved in fitness. Furthermore, we evaluated bacterial growth in C medium. The EH41 strain showed a significant slow growth when compared with Ec472/01 (S1 Fig).

### Gene co-expression networks (GCN) for EH41 and Ec472/01

**Fig 4:**
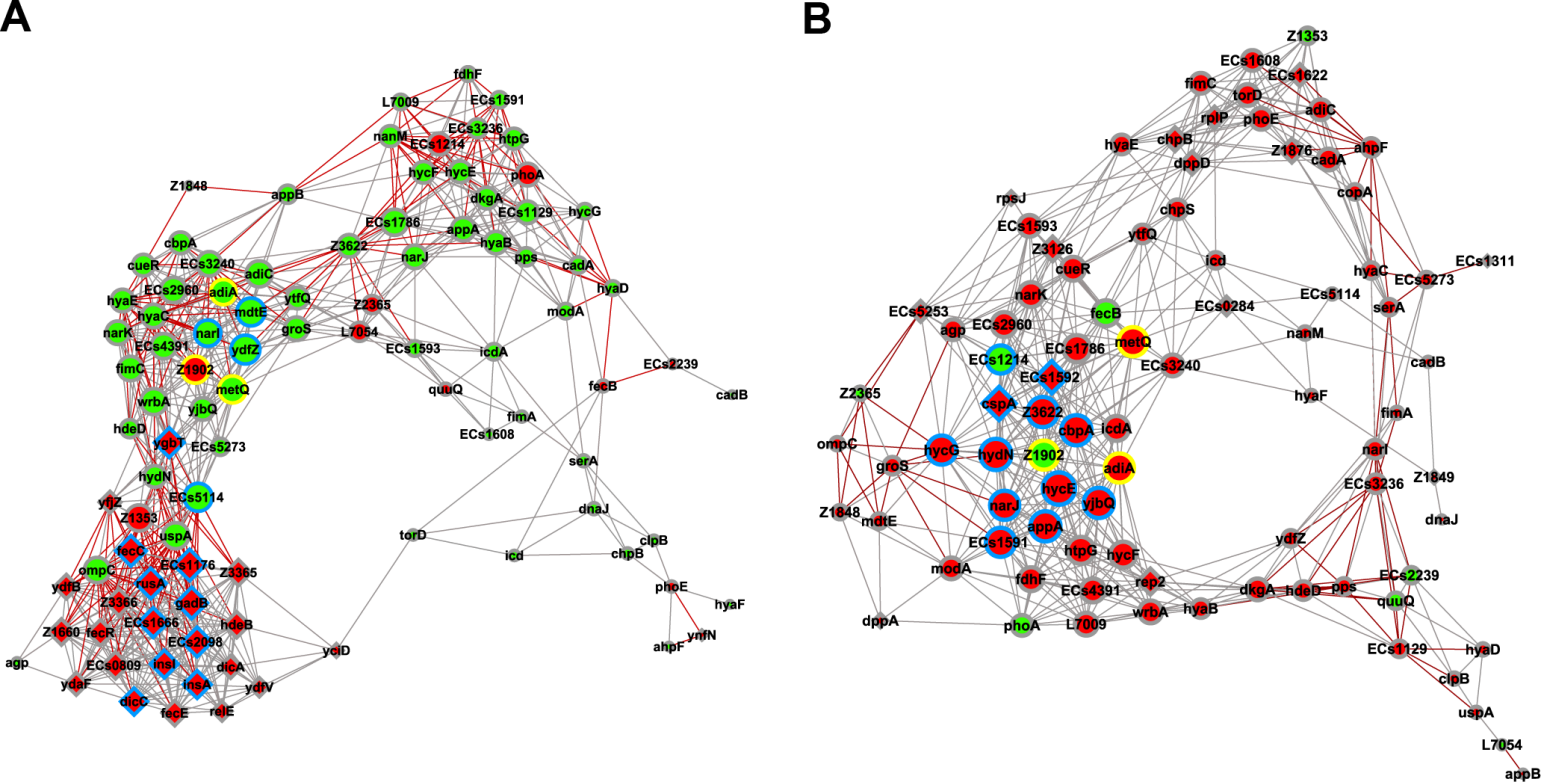
DE and EE gene co-expression networks (GCNs) for STEC strains. GCNs for EH41 and Ec472/01 are depicted in (A) and (B) respectively. The most relevant hubs (high number of gene-gene links) are graphically represented by the proportionally larger nodes. Positive or negative Pearson’s coefficients—indicating positive or inverse covariation between gene-pairs—are represented by gray or red lines respectively. Nodes in red or green indicate hyper or hypo expressed genes, respectively. Circle or diamond nodes indicate DE or EE genes respectively. Node borders in blue indicate hub genes; node borders in yellow indicate common hubs between the two GCNs.

This GCN analysis considered just the DE and EE genes obtained from the comparison between the transcription profiles of EH41 and Ec472/01 strains grown in C medium. The two networks were constructed considering only the transcripts codifying for known or putative proteins: 103 transcripts for EH41 and 89 transcripts for Ec472/01 (Table 2, Fig 4). A list of the most relevant hubs, i.e. those presenting high numbers of gene-gene links, and their biological function appears in Table 3.

**Table 2:** Gene co-expression network characteristics for each group.

	Network		
Group	No. nodes	No. links	Cut-off (|*r*|≥)
EH41	96	737	0.90
Ec472	87	542	0.90
Caco2-EH41	83	650	0.94
Caco2-Ec472	113	684	0.99
Caco2-Control	135	528	0.98

**Table 3:** Hubs in EH41 and Ec472/01 networks.

Gene	EH41	Ec472/01	Gene function
*ECs1176*^*a*^	**26**	NE	host-nuclease inhibitor protein Gam; phage recombination [20]
*gadB*^*a*^	**26**	NE	stomach acid resistence [23]
*fecC*^*a*^	**25**	NE	transmembrane protein involved in the ferric citrate transport [31]
*rusA*^*a*^	**25**	NE	endonuclease RUS; phage recombination [21]
*ECs1666*^*a*^	**24**	NE	transposase; phage recombination [22]
*ECs2098*^*a*^	**24**	NE	glutamate decarboxylase isozyme; acid stress adaptation [23]
*insA*^*a*^	**22**	NE	transposase; phage recombination [32]
*insI*^*a*^	**22**	NE	IS30 transposase; phage recombination [33]
*dicC*^*a*^	**21**	NE	DNA-binding transcriptional regulator [34]
*ygbT*	**24**	NE	CRISP-associated protein Cas1 [24, 25]
*ydfZ*	**25**	9	Selenoprotein; involved in survival in the host oxidative attack [35, 36]
*mdtE*	**24**	8	multidrug efflux transporter; increases multidrug resistance [29]
*ECs5114*	**24**	4	pH stress sensor; acid stress adaptation [28]
*narI*	**22**	11	membrane subunit of NarGHI complex involved in nitrate reduction [30]
*metQ*	**23**	**21**	DL-methionine transporter subunit [27]
*Z1902*	**23**	**21**	phage recombination [37]
*adiA*	**21**	**21**	biodegradative arginine decarboxylase [26, 38]
*cspA*	NE	**23**	prevention of RNA secondary structure formation [39]
*ECs1592*	NE	**22**	head portal protein; bacteriophage DNA packaging machine
*hycE*	17	**24**	formate hydrogenlyase subunit 5; electron transfer [40]
*hydN*	17	**23**	electron transport protein HydN [41]
*Z3622*	18	**22**	recombinase; transposition of *stx* in *E*. *coli* [42]
*appA*	19	**22**	phosphoanhydride phosphorylase; phytase
*cbpA*	17	**22**	curved DNA-binding protein; stationary phase-specific nucleoid protein [43]
*yjbQ*	18	**22**	thiamin phosphate synthase [44]
*ECs1591*	11	**21**	prohead protease
*hycG*	10	**21**	hydrogenase 3 and formate hydrogenase complex, HycG subunit; electron transfer [40]
*narJ*	19	**21**	chaperone subunit of nitrate reductase; involved in respiratory process [45]
*ECs1214*	17	**20**	antirepressor protein [46]

^a^Hubs in the *dicA* transcriptional module; number of links in bold indicates a hub gene in the network; NE: genes not expressed by STEC Ec472/01 or EH41

### EH41 network

**Fig 5:**
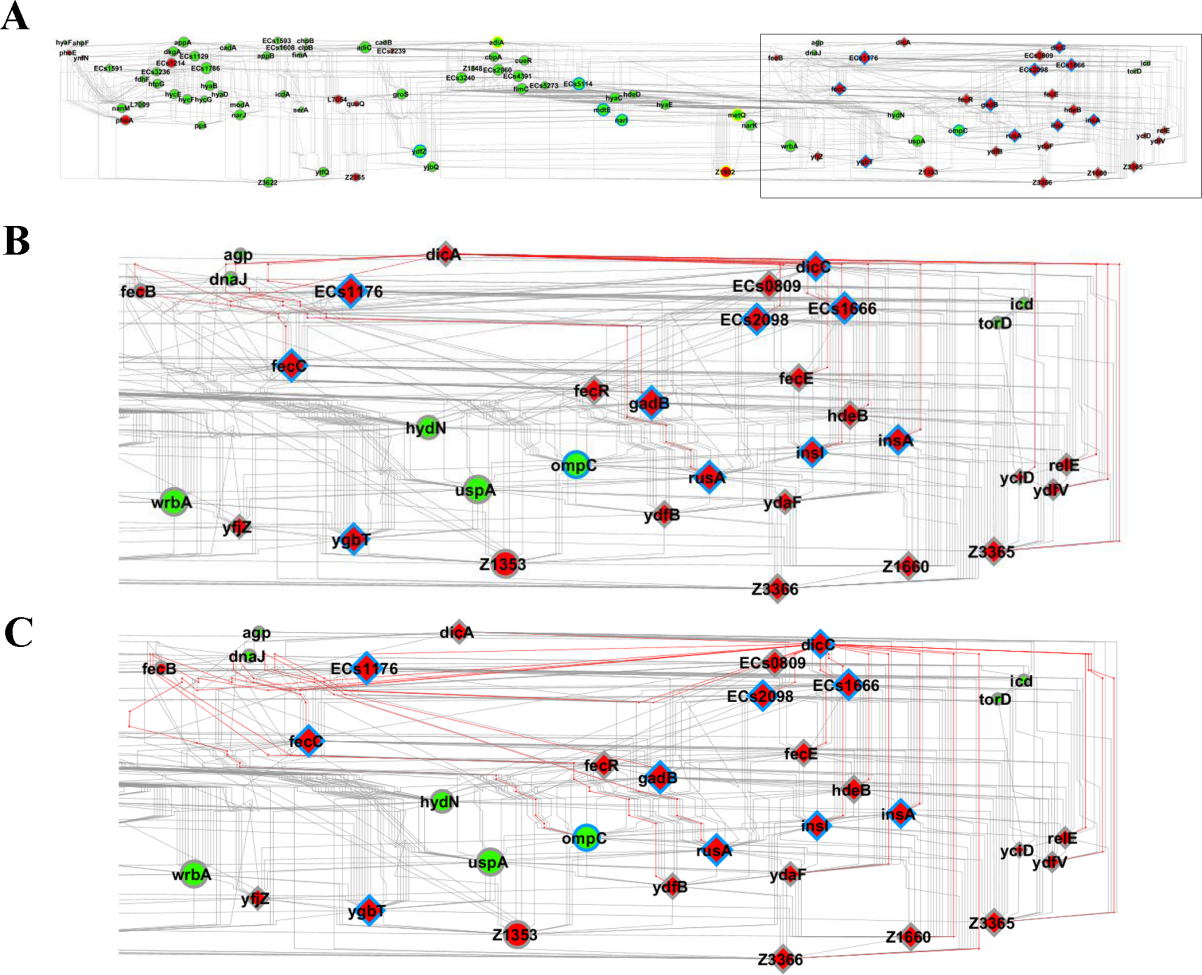
Hierarchical structure of the EH41 network. Node hierarchical arrangement represents the main direction within a network (A). In (B) and (C) the EE transcriptional module is displayed in detail. Links in red represent the first node connections, centered in *dicA* (B) or in *dicC* (C). Nodes in red or green indicate hyper or hypo expressed genes, respectively. Circle or diamond nodes indicate DE or EE genes respectively. Node borders in blue indicate hub genes; node borders in yellow indicate common hubs between EH41 and Ec472/02 GCNs.

The EH41 GCN (Fig 4A) revealed a major transcriptional module constituted by genes exclusively expressed (EE) in STEC EH41 and containing most of the hubs in this network (10 out of 16). This module presents positive interaction between its constituent EE genes (diamond nodes) but negative interaction with the network’s DE genes (circle nodes). Moreover, the EH41 hierarchical network (Fig 5) showed that the transcriptional regulators *dicA* and *dicC* are the first and the second hierarchical genes in the EE transcriptional module, here named *dicA* module. Moreover, *dicC* is linked, in the first level, with almost all the EE genes (20 out of 23, where 8 are hubs), whereas *dicA* is linked with 16 EE genes, of which nine are hubs, including *dicC* (these links are indicated by red lines in Fig 5B and 5C). Here is important to mention that the genes *dicA* and *dicC* are part of the Qin prophage [17] and that DicA is homologous to the virulence regulators RovA and SlyA [18, 19]. This issue will be discussed further.

The list of hubs and their respective biological functions is presented in Table 3. The *dicA* module encompasses 27 genes: 23 EE genes, of which nine are hubs, and four are DE genes, of which only one is a hub. In this module seven EE hubs may be involved in bacterial virulence (*dicC*, *ECs1176*, *rusA*, *ECs1666*, *ydfZ*, *insA* and *insI*) [20–22] and two EE hubs are associated with acid stress adaptation to survive in the host (*gadB* and *ECs2098*) [23].

The second major module encompasses 24 DE genes, of which seven are hubs and only one is an EE gene (*ygbT*). This EE hub codifies for a CRISP-associated protein related to antivirus immunity and DNA repair [24, 25]. Three DE genes are also hubs in the Ec472/01 network: *adiA* is involved in acid stress adaptation [26]; *metQ* is involved in ABC-type transporter in *E coli* [27]; and *Z1902*, which codifies for prophage head-tail adaptor. Two other hubs (*ECs5114* and *mdtE*) are involved in bacterial survival in the host [28, 29]. The last hub, *narI*, codifies for a membrane anchor subunit of NarGHI complex involved in electron transfer [30]. It is interesting to note that only one DE hub, *Z1902*, is hyperexpressed in EH41.

### Ec472/01 network

The Ec472/01 GCN (Fig 4B) has 15 hubs (Table 3). Ec472/01 network contains only one major transcriptional module encompassing 43 genes (37 DE genes and six EE genes). Only three out of 13 DE hubs appear to be involved in virulence: *adiA*, a common hub with EH41 network, is involved in acid stress adaptation [26]; *ECs1214*, codes for an antirepressor protein associated to a superinfecting STEC [46]; and Z3622, which is a phage-derived gene [42]. Two out of six EE genes are hubs—*cspA* and *ECs1592* –codifying, respectively, for an RNA chaperone [39] and for a head portal protein. The remaining hubs are involved in various metabolic processes, such as electron transfer, amino-acid synthesis or transcription.

### PCR detection of the *dicA* module EE genes using a panel of STEC O113:H21 strains

We used PCR for detecting the presence of nine EH41 EE genes—*insI*, *ECs2098*, *dicA*, *dicC*, *fecC*, *gadB*, *Ecs1176*, *insA* and *rusA*—of the *dicA* module in a panel constituted by Ec472/01, one Brazilian STEC strain isolated from beef meat and nine other Brazilian STEC strains isolated from the animal reservoir (S1 Table). This analysis revealed that five genes (*dicA*, *fecC*, *Ecs1176*, *insA* and *rusA*) are absent in all these strains. Two genes—*ECs2098* and *gadB*—were detected in all strains (Table 4).

**Table 4:** PCR gene detection in a panel of STEC O113:H21 strains.

			PCR gene detection^a^								
Strain	MLST	Source	*insI*	*ECs2098*	*dicA*	*dicC*	*fecC*	*gadB*	*Ecs1176*	*insA*	*rusA*
EH41^b^	820	HUS	+	+	+	+	+	+	+	+	+
Ec472/01^c^	ND	Bovine feces	+	+	-	-	-	+	-	-	-
226/1	846^e^	Bovine feces	+	+	-	+	-	+	-	-	-
Ec670/05	846^e^	Bovine feces	+	+	-	-	-	+	-	-	-
Ec254/01	997	Bovine feces	-	+	-	-	-	+	-	-	-
Ec226/04	223^d^	Bovine feces	-	+	-	-	-	+	-	-	-
Ec503/05	ND	Goat feces	-	+	-	-	-	+	-	-	-
Ec182/04	ND	Buffalo feces	+	+	-	+	-	+	-	-	-
Ec624/05	ND	Bovine feces	+	+	-	+	-	+	-	-	-
Ec684/04	ND	Bovine feces	-	+	-	-	-	+	-	-	-
Ec253/02	997	Bovine feces	-	+	-	-	-	+	-	-	-
Ec784	997	Beef meat	-	+	-	-	-	+	-	-	-

^a^Symbols: (+) for positive or (-) for negative PCR product

^b^strain EH41 express these genes

^c^strain Ec472/01 does not express these genes

ND, not determined; same clonal group of the STEC strains isolated from an Argentine HUS-patient^d^ and from a Germany patient with diarrhea^e^ [11]

### Transcriptional analyses of Caco-2 cells after interaction with EH41 and Ec472/01 STEC strains

Gene expression comparative analyses were performed for Caco-2 cells after 3h of interaction with EH41 (Caco2-EH41) or with Ec472/01 (Caco2-Ec472). The comparison between Caco2-EH41 or Caco2-Ec472 with Caco2-Control (no bacterial interaction) revealed 91 DE GO (Gene Ontology)-annotated genes for Caco2-EH41 group and 136 DE GO-annotated genes for Caco2-Ec472 group (Table 1). All these DE genes were analyzed through Venn diagram. The result showed that 66 hyperexpressed genes and six hypoexpressed genes are common for both comparisons (S2 Fig). Consequently, the functional profiles for the DE genes of Caco2-EH41 and Caco2-Ec472 were found to be very similar (S3 Fig).

### GCN analysis of Caco-2 cells after interaction with STEC strains

**Fig 6:**
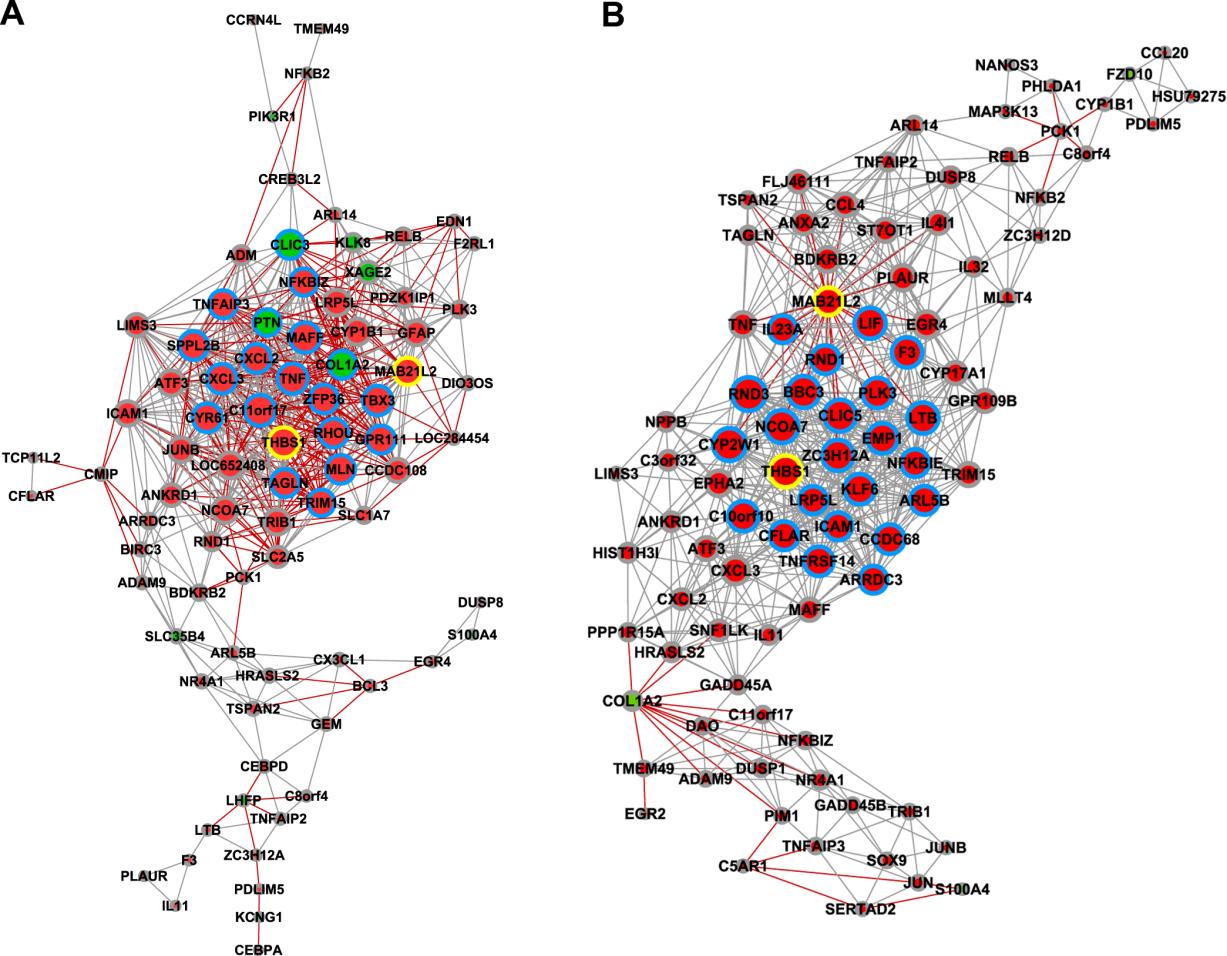
DE gene co-expression network (GCN) for Caco-2 cells after interaction with STEC strains. GCNs for Caco-2 interacting with EH41 or with Ec472/01 are shown in (A) and (B) respectively. Hubs are graphically represented by the proportionally larger nodes. Positive or negative Pearson’s coefficients are indicated by gray or red lines respectively. Nodes in red or green indicate hyper or hypo expressed genes, respectively. Blue node border indicates a hub gene; yellow node border indicates a common hub between the two GCNs.

**Fig 7:**
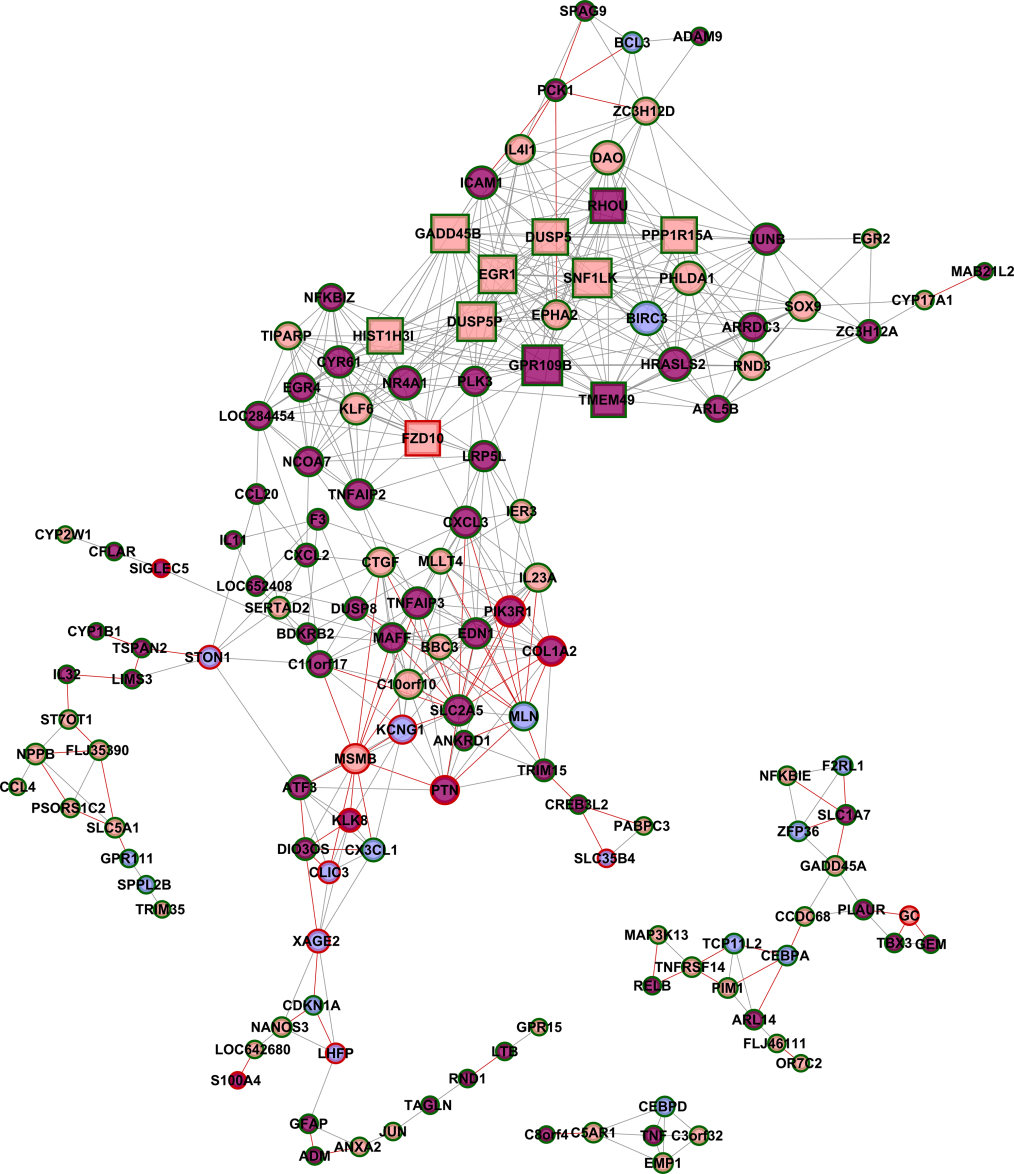
DE gene co-expression network (GCN) for Caco-2 cells control group. Caco2-control network was constructed considering all DE genes obtained from two comparisons: Caco-2 with EH41 X Caco-2 control and Caco-2 with Ec472/01 X Caco-2 control. Hubs (square nodes) are graphically represented by the proportionally larger nodes. Positive or negative Pearson’s coefficient is indicated by gray or red lines respectively. Nodes in blue, pink or purple indicate, respectively: DE genes present only in Caco-2 interacting with EH41, or with Ec472/01 or common for the two groups. Node borders in green or red indicate hypo or hyper expressed genes in the control group.

Three gene co-expression networks were constructed for DE genes (Table 2): i) for Caco2-EH41 (Fig 6A); ii) for Caco2- Ec472 (Fig 6B); and iii) for Caco2-Control (Fig 7). The control network was based on all DE genes obtained in the two former comparisons (155 genes, S2 Fig). A list of the most relevant hubs according to the number of gene-gene links appears in Table 5.

**Table 5:** Hubs in Caco-2 cells with EH41 or with Ec472/01 and control networks.

	Number of links^a^			
Gene	EH41	Ec472/01	CT	Gene biological function
*C11orf17*	**34**	9	10	aliase AKIP1; involved on the NF-kappa-B activation cascade [51]
*TNF*^*d*^	**33**	20	4	codifies for proinflammatory cytokines family [78]
*CXCL3*^*d*^	**32**	23	15	codifies for chemoatractant for neutrophils; involved in inflammation [47]
*CXCL2*^*d*^	**31**	14	7	codifies for chemoatractant for neutrophils; involved in inflammation [50]
*SPPL2B*	**31**	0	2	involved in cytokine expression in the innate and adaptive immunity pathways [49]
*ZFP36*	**31**	0	4	negative regulator of cytokine production [53]
*NFKBIZ*	**29**	9	11	involved in induction of inflammatory genes activated through TLR/IL-1 receptor signaling [48]
*PTN*	**28**^**b**^	1	12	encodes a pleiotrophin; induces the production of inflammatory cytokines, including TNF-alfa, IL-1b and IL-6 [52]
*COL1A2*	**30**^**b**^	12	13	encodes the pro-alpha2 chain of type I collagen [60]
*TRIM15*	**28**	18	6	a member of the tripartite motif (TRIM) family; involved in regulation of immune signaling pathways [55]
*THBS1*	**33**	**29**	0	adhesive glycoprotein [61]; involved in phagocytosis [62]
*CLIC3*	**30**^**b**^	0	6	CLIC family proteins have been associated to macrophage activation [54]
*CYR61*	**31**	0	15	Involved in apoptosis [56]
*TAGLN*	**30**	11	2	overexpression of TAGLN dismishes cell proliferation and improves cell apoptosis in colorectal carcinoma cells [57]
*TNFAIP3*^*d*^	**30**	8	15	A20 (aliase), major antiapoptotic protein (via TNF) in the intestinal epithelium [58]
*RHOU*	**31**	0	**18**	involved in the regulation of cell morphology and cytoskeletal organization [79, 80]
*MLN*	**31**	0	12	involved in the regulation of interdigestive gastrointestinal motility [81, 82]
*GPR111*	**30**	0	2	members of the superfamily of human G protein-coupled receptors
*MAB21L2*	**29**	**24**	1	involved in cell growth
*MAFF*	**33**	17	13	small MAF transcription factor inducted by interleukin 1 beta (IL1B) and a weaker upregulated by TNF [83]
*TBX3*	**32**	0	3	transcriptional factor
*LTB*	4	**28**	2	codifies for cytokine that binds to LTBR/TNFRSF3; involved in cytokine production [66, 68]
*LIF*^*d*^	0	**28**	0	encodes a pleiotropic cytokine
*TNFRSF14*	0	**26**	4	Several members of the TNFSF are closely associated with inflammatory bowel disease [67]
*ICAM1*^*d*^	25	**24**	16	involved in innate immune response induced by enteroinvasive bacteria [63, 64]
*IL23A*	0	**24**	12	codifies for cytokine; induced by LPS [69, 84]
*NFKBIE*	0	**24**	3	codifies for a protein belonging to NF-kappaB inhibitors proteins family [65]
*BBC3*	0	**29**	9	aliase PUMA; encodes a member of the BCL-2 family involved in apoptosis [76]
*PLK3*	13	**27**	13	involved in apoptosis and stress responses [75]
*EMP1*	0	**27**	4	involved in apoptosis and cell adhesion [74]
*KLF6*	0	**27**	15	involved in apoptosis; has been shown to be induced by bacterial toxins [72]
*CFLAR*^*d*^	2	**25**	2	aliase CASP8AP1; antiapoptosis regulator protein; acts as an inhibitor of TNFRSF6 mediated apoptosis [73]
*RND3*	0	**31**	12	negative regulator of cytoskeletal organization [85]
*CLIC5*	0	**29**	0	Chloride intracellular channel 5; cytoskeleton organization [86]
*RND1*	15	**25**	0	controls rearrangements of the actin cytoskeleton [87]
*NCOA7*	26	**30**	13	involved in human autophagy system [70]
*ZC3H12A*	4	**28**	7	Regnase-1 or MCPIP1 (aliases); involved in inducing and suppressing inflammatory responses [71]
*ARL5B*	7	**25**	10	protein transport regulator—including STX—along the endosome to Golgi trafficking in HeLa cells [77]
*CYP2W1*	0	**30**	1	involved in catalytically activate compounds to cytotoxic products [88]
*ARRDC3*	11	**24**	12	Ubiquitination process [89]
*F3*	3	**27**	5	F3 initiates the blood coagulation cascades
*LRP5L*	27	**24**	14	Codifies a low density lipoprotein; involved in signal transduction [90]
*CCDC68*	0	**26**	3	colorectal tumor associated protein [91]
*C10orf10*	0	**27**	13	chromosome 10 open reading frame 10; aliase DEPP, Fseg
*DUSP5P*	0	1	**22**	hyperexpressed in tumor cells [92]
*PPP1R15A*	0	10	**19**	aliase GADD34;hyperexpressed in human colon epithelial cells [93]
*FZD10*	0	4	**18**^**c**^	hyperexpressed in colorectal carcinoma [94]
*GADD45B*	0	7	**21**	hyperexpressed in colorectal carcinoma [95]
*DUSP5*	0	1	**19**	hyperexpressed of DUSP5 suppress the growth of several types of human cancer cells [96]
*TMEM49*	1	7	**18**	aliase VMP1; VMP1-dependent autophagy in colorectal cancer cells [97]
*EGR1*	0	1	**21**	histone acetyltransferase binding protein family; involved in claudin-3 transcription, a tight junction protein, in Caco-2 cells [98]
*HIST1H3I*	0	12	**19**	a member of the histone H3 family [99]
*SNF1LK*	0	14	**22**	Salt-inducible kinase 1 belongs to the AMP-activated protein kinase (AMPK) family [100]
*GPR109B*	2	17	**23**	G protein-coupled receptor 109B; involved in the activation of ERK1/2 MAP kinase pathway [101]

^a^Number of links in bold indicates a hub gene in the network

^b^genes hypoexpressed in Caco-2 cells with EH41

^c^genes hyperexpressed in Caco-2 control group

^d^TNF signaling pathway (KEGG, map04668)

### Caco2-EH41 network

The network analysis revealed 21 hubs, all belonging to a single major transcriptional module. All these hubs present many negative and positive gene-gene interactions, thus reflecting gene expression assynchrony (Fig 6A**)**. It is noteworthy that 13 out of 21 hubs are involved in innate immunity. Eight of these hubs are involved in inflammatory response (*TNF*, *SPPL2B*, *ZFP36*, *NFKBIZ*, *PTN*, *CXCL3*, *CXCL2* and *C11orf17*) [47–53]. The hubs *CLIC3* and *TRIM15* are respectively involved in macrophage activation [54] and immune signaling pathways [55]. Finally, three hubs are involved in apoptosis (*CYR61* and *TAGLN*) [56, 57] or antiapoptosis (*TNFAIP3*) [58].

Additionally, two other hubs could also be involved in immune response, *COL1A2* and *THBS1*. The first encodes a cellular matrix protein that can be stimulated through TLR4 by *E*. *coli* and induces a chronic inflammatory state in murine colitis [59, 60]. The former codifies for an adhesive glycoprotein and its deficiency is associated with decreased phagocytosis and possibly bacterial clearance [61, 62]. The remaining six hubs are involved in cytoskeletal organization, signaling and cell growth or codifies a G protein-coupled receptor and transcription factors. Two hubs—*THBS1* and *MAB21L2*—are also hubs in Caco2-Ec472 network and *RHOU* is likewise hub in Caco2-Control network (Table 5).

### Caco2-Ec472/01 network

This network contains 25 hubs, all belonging to a single major transcriptional module. All genes in this module present positive interaction, except for *MAB21L2*, involved in signaling and cell growth: all their links have negative co-variation coefficient (Fig 6B). This GCN showed that half of the hubs (14 out 25) are involved in immune response. Six hubs are related to inflammatory response (*LTB*, *LIF*, *TNFRSF14*, *NFKBIE*, *IL23A* and *ICAM1*) [63–69], two are involved in autophagy (*NCOA7* and *ZC3H12A*) [70, 71], and one in phagocytosis (*THBS1*) [62]. Additionally, five hubs (*BBC3*, *PLK3*, *EMP1*, *KLF6* and *CFLAR*) [57, 72–76] are associated to apoptosis (Table 5 displays the detailed description of these hubs functions).

Other four hubs also have interesting features: three of these hubs are involved in cytoskeleton organization (*CLIC5*, *RND1* and *RND3*) and one (*ARL5B*) codifies for a protein transport regulator. In HeLa cells this protein is involved in Shiga toxin transport along the endosome to Golgi trafficking [77]. The remaining hubs are involved in other biological functions such as signaling, metabolism and ubiquitination.

### Caco2-Control network

The Caco2-Control network analysis (Fig 7) revealed that all hubs belong to a single major transcriptional module and present positive gene-gene interactions. The majority of the hubs (9 out of 11) are involved in cell growth control (seven hubs) and epigenetics (two hubs). Other two hubs are related to regulation of cell morphology and ion transport (Table 5).

### Caco-2 cells SEM images

**Fig 8:**
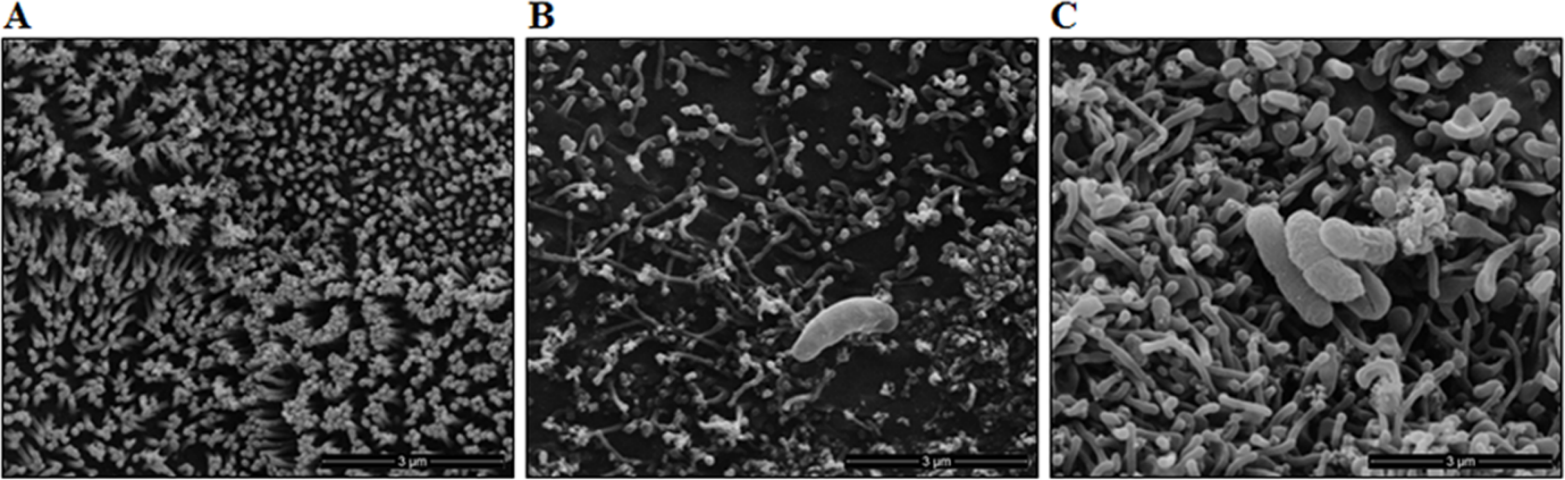
SEM visualization of Caco-2 cells after 3h of interaction with STEC strains. The images depict the Caco-2 cells control (A) and cells interacting with EH41 (B) or Ec472/01 (C).

Fig 8 shows SEM images of Caco-2 cells after their interaction with EH41 or Ec472/01 strains. These images revealed that both strains are capable of inducing cell morphology alterations. However, the interaction with EH41 leads to a severe microvilli loss (Fig 8B), whereas Ec472/01 induced only modest microvilli loss (Fig 8C), but significant microvilli morphology change (elongation, orientation) when compared to Caco-2 control cells (Fig 8A).

### qPCR validation of microarray data

#### STEC group

Three hyperexpressed (*phoA*, *phoE* and *EcS1174*) and two hypoexpressed (*hycE* and *hycG*) genes were selected for qPCR analysis. The fold-changes for each gene—comparing EH41 versus Ec472/01 groups’ average of relative gene expression—confirmed DNA microarray gene expression results (S4 Fig).

#### Caco-2 cell group

The same experimental design was followed: three hyperexpressed (*BIRC3*, *CCL20* and *ZC3H12A*) and two hypoexpressed (*PTN* and *STON1*) genes were selected. The fold-changes for each gene—comparing Caco2-EH41 versus Caco2-Ec472 groups’ average of relative gene expression—confirmed DNA microarray gene expression results (S5 Fig).

## Discussion

The association of O113:H21 STEC strains with hemolytic uremic syndrome (HUS) has been reported in several countries [11]. In Brazil O113:H21 strains are commonly found in the animal reservoir but, so far, were not isolated from HUS patients [7, 8]. Interestingly, some Brazilian O113:H21 strains and strains isolated from HUS patients in Argentina belong to the same clonal group [11]. Several studies have been done in STEC strains isolated from animal and environmental sources aiming at identifying HUS-related virulence genes and characterizing the pathogenic potential of these strains. However there is no specific virulence genetic profile that enables one to distinguish between the pathogenic and environmental strains [6, 11, 102]. In this study we were able to show genomic and phenotypic differences between two O113:H21 STEC strains: EH41, a reference strain isolated from HUS patient in Australia, and Ec472/01, isolated from cattle feces in Brazil. We also characterized the differential enterocyte response after exposure to EH41 or Ec472/01.

First we investigated if Caco-2 cell soluble mediators could increase the expression of STEC virulence genes in EH41 and Ec472/01 bacteria. Comparative global gene expression analyses revealed, for both strains, that the expression of virulence genes was not significantly different: only one per cent of the transcripts were differentially expressed. Furthermore, the majority of these transcripts was not yet functionally characterized, as in EH41, or involved in metabolic processes, as in Ec472/01 (S4 and S5 Tables). Comparatively, in EHEC, more than 1,400 genes–most of them related to virulence—were differentially expressed after culturing in enterocyte conditioned medium [16]. These results indicate that EH41 and Ec472/01 virulence genes are not modulated by soluble mediators, i.e. their virulence phenotypes were largely set before infecting the human host.

Therefore we conducted a comparative transcriptomic analysis between EH41 and Ec472/01 cultured in C medium. The results revealed that these two strains have different gene expression profiles. We found important differences in these profiles: i) 82 out of 97 DE transcripts are hypoexpressed in EH41 and almost half of these genes are involved in metabolic process (Figs 2 and 3); ii) in EH41 10 out of 29 EE genes are prophage-derived genes; iii) in Ec472/01 only seven out of 35 EE genes are functionally characterized an all related to metabolic or transcriptional control processes (S6 Table).

It is interesting to note that in EH41 six of the EE genes belong to the Qin prophage [17]: *dicA*, *dicC*, *relE*, *ynfN*, and two not yet functionally characterized. It is well known that cryptic prophages provide multiple adaptative advantages for the bacteria, such as antibiotic resistance, oxidative and acid stresses tolerance, enabling survival in adverse environmental conditions [103]. The *dicA* is an *E*. *coli* transcriptional regulator acting as a temperature sensor repressor for bacterial growth and, when mutated, is complemented by the adjacent gene *dicC* [34, 104]. Additionally, DicA shares significant amino acid sequence similarity with the DNA-binding domains of RovA and SlyA, which are regulators of pathogenic genes in *Yersinia* and *Salmonella*, respectively [17]. These two proteins regulate a wide range of physiological processes involved in survival, stress adaptation, and virulence [18, 19]. In *Salmonella*, SlyA regulates virulence factors necessary for environmental adaptation and survival in mice [105]. In *E*. *coli* SlyA induces a cryptic *hlyE* gene (aliase *clyA*), which encodes for hemolysin E [106].

Hierarchical analysis of the EH41 network showed that *dicA* is the first hierarchical gene in the EE module and encompasses 22 out of the 24 EE genes in the network (Fig 5). The *dicA* transcriptional module is distinctive of the EH41 network and seems to be very important since it encompasses 11 out of the 16 network hubs. Moreover, most of these hubs are prophage-derived genes, including *dicC*, or involved in acid stress adaptation, what indicates that the *dicA* module could well be related to EH41 virulence.

The other two modules in EH41 network (Fig 4A) encompass only DE genes—many of them involved in metabolic process. The majority of these genes are hypoexpressed in EH41, except for the hub *Z1902*, which is a prophage-derived gene. It is interesting to note that *metQ*—also a hub in Ec472 network, but hypoexpressed in EH41 –codifies for a DL-methionine transporter subunit and may be involved in bacterial growth efficiency. *Streptococcus pneumoniae metQ* and *metEF* mutants show a decreased growth in methionine restricted conditions [107]. Here we confirmed that EH41 has a diminished growth in C medium when compared with Ec472/01 (S1 Fig).

Altogether, EH41 GCN analysis indicates that *dicA* positively regulates other EE genes (probably virulence genes) in the *dicA* transcriptional module. Here it is worth to note that the *dicA*, *B*, *C* gene family exerts a negative regulation on bacterial growth [34, 104]. These two regulatory roles of *dicA* could represent an adaptation for improving long-term bacterial survival in the enteric environment. Indeed, there are reports of long-term STEC shedding, i.e. well after the symptoms are resolved. The median duration of shedding has been shown to be 20 days; however, some patients were STEC PCR-positive up to 9 months after symptoms disappearance [108, 109].

Ec472/01 network presented just six EE genes which are scattered in a few transcriptional modules (Fig 4B). In this network only two out of 15 hubs are EE genes, but all these hubs belong to a single main transcriptional module. As mentioned before, most of the DE genes are hyper-expressed in Ec472/01 and probably involved in bacterial growth (S6 Table). Five hubs may be related to virulence: two are phage-derived genes, one is involved in acid stress adaptation, one codifies for a recombinase and the last codifies for an antirepressor protein. Recombinase and antirepressor proteins are associated with acquisition and expression of bacteriophage-virulence genes [42, 46, 110].

Our results clearly indicate that *dicA* transcriptional module, and especially the *dicA* gene, contributes to the distinctive phenotypic difference between EH41 and Ec472/01. Hence, we investigated the presence of *dicA* and other eight EH41 EE hub genes in a STEC panel. We selected STEC strains from different clonal groups, including strains belonging to same clonal group of the STEC strains isolated from an Argentine HUS-patient and from a German patient with diarrhea [11]. The results revealed that all STEC strains isolated from bovine, goat or buffalo feces, or from beef meat, lack *dicA* and four other genes present in EH41 (Table 4). This result indicates that STEC strains isolated from animal reservoirs do not have the complete gene repertoire to cause severe diseases, such as HUS. Hereafter, it would be necessary to extend this investigation to other STEC strains isolated from patients with HUS, hemorrhagic colitis, and diarrhea, as well as from animal and environmental sources. In this study, we could not test other O113:H21 STEC strains isolated from patients because: i) this serotype has not yet been isolated from HUS patients in Brazil, and ii) nowadays for biosafety reasons HUS-associated STEC strains from other countries is hard to obtain. Nevertheless, it is utterly important to undergo this kind of investigation in order to provide biomarkers for identifying STEC strains capable of causing HUS and related severe diseases.

The Caco-2 cell response after three hours of interaction with STEC was investigated by GCN analysis for DE genes. Distinct enterocyte responses to EH41 and Ec472/01 strains in DE networks are clearly evidenced by the pronounced differences in network topology (Fig 6A and 6B). Caco2-EH41 GCN reflects global gene expression dysregulation, since this network has many positive and negative gene-gene covariation coefficients in the major module. Conversely, in Caco2-Ec472/01 and Caco2-Control networks most of the nodes have positive links and just a few genes–corresponding to the nodes at the transcriptional module border or nodes connecting two modules—have negative links. This is what is expected in normal cell functioning or just before the health-disease transition [111–113]. Nodes with positive links tend to cluster together, while nodes with negative links usually act as bridges between clusters of positively linked nodes [112]. This result suggests that EH41 is capable to induce, after three hours of cell-bacteria interaction, an intense genomic dysregulation in Caco-2 cells.

The majority of the hubs in Caco2-EH41 GCN were found to be related to inflammatory/innate immune response (Table 5). It is possible to infer, based on KEGG data, that several hubs in this network are involved in the activation of inflammatory response and neutrophil recruitment via TNF signaling pathway (Table 5). Actually, *CXCL2* and *CXCL3* (aliase *GROg*) codify for chemoattractants involved in neutrophil migration [47, 50] and, TNF, an inflammatory cytokine. Additionally the hub PTN encodes a pleiotropin and induces production of inflammatory cytokines, such as TNF, IL-1b and IL-6 [52]. In mice, CXCL2 is involved in neutrophil migration into the kidney after exposure to EHEC O157:H7 (*E*. *coli* possessing *stx*/*eae* genes and associated with HUS) virulence factors [50]. The increased production of neutrophil chemoattractants such as IL-8, Stx-mediated ERK1/2 activation promotes inflammation and the systemic uptake of Stx, leading to the onset of HUS [114]. Moreover, it was described that flagellin is the major EHEC determinant contributing to chemokine production in human intestinal epithelium [115], suggesting that Stx is not a major participant in promoting intestinal inflammation. It is possible that in EH41 Stx exacerbates epithelial inflammation induced firstly by other virulence factors.

This scenario did not occur in Caco-2 cells during interaction with Ec472/01. Only one third of the hubs are involved in innate immunity and enrichment analysis based on KEGG molecular pathways showed that three hubs are related to TNF signaling pathway: i) cell adhesion (*ICAM1*); ii) inflammatory cytokine (*LIF*) and; iii) *CFLAR* that codifies an apoptosis inhibitor regulator [116]. Additionally, both networks have hubs involved in cystoskeletal organization.

Scanning electron microscopy (SEM) images revealed microvilli loss after Caco-2 interactions with EH41 or with Ec472/01 (Fig 8). Epithelial cell morphology alterations are clearly more severe after exposure to EH41, including brush border and microvilli destruction. Microvilli establish an electrostatic barrier to microbial adhesion [117] and, therefore, EH41-induced microvilli destruction may contribute to the enhanced persistence of this strain in the enteric environment.

There is evidence that acute inflammation plays a role in the development of HUS. Patients with HUS demonstrate a rise in c-reactive protein, neutrophilia and an increase in circulating proinflammatory cytokines, indicating that the impact of hemorrhagic colitis may be important for the subsequent development of severe complications, such as HUS and encephalopathy [78]. Moreover, Stx probably is not the central factor involved in enteric inflammation, but together with others bacterial effectors contribute to promote inflammation and neutrophil migration.

In conclusion, we used a gene co-expression network (GCN) analysis to investigate two STEC strains—one associated to HUS (EH41) and another isolated from cattle (Ec472/01)—and also the enterocyte response after bacterial interaction. Comparative STEC GCN analysis revealed that STEC HUS-associated has a distinctive *dicA* transcriptional module and majority of the genes in others modules—possible related to bacterial growth—are hypoexpressed. This fact may be related to bacterial adaptation to survive for long period in intestinal environment and consequently cause severe disease in human. The PCR detection of nine hub genes belonging to *dicA* module in EH41 network indicated that STEC strains isolated from animal reservoirs could not have complete gene repertoire to cause severe disease, such as HUS. Moreover, comparative Caco-2 cells GCN analysis indicated that STEC HUS-associated induces pronounced inflammation response and gene network dysregulation. Finally, *dicA* and other four genes (*fecC*, *insA*, *rusA* and *ECs1176*) may be used as molecular markers to distinguish between HUS-associated O113:H21 STEC strains and other strains isolated from animal or environmental sources. This characterization is important since some O113:H21 STEC strains isolated from animal sources in Brazil belong to the same clonal group of STEC strains isolated from Argentine HUS-patients or from animal sources.

## Supporting information

S1 Fig Determination of bacterial concentration by optical density. Bacterial concentrations for STEC EH41 and Ec472/01 strains grown in conditioned medium for 3 hours.
(TIF)

S2 Fig Comparative analyses of Caco-2 gene expression profiles. Venn diagram analysis of DE genes obtained from two comparisons: (A) Caco-2 with EH41X Caco-2 control or Caco-2 with Ec472/01 X Caco-2 control; (B) Venn diagram analysis of hyper and hypo expressed genes between the two groups.
(TIF)

S3 Fig Functional profile analyses of DE genes for Caco-2 cells. Pie charts of DE genes obtained from two comparative analyses: Caco-2 with EH41 X Caco-2 control (A) or Caco-2 with Ec472/01 X Caco-2 control (B). Functional categories are identified by roman numerals as follows: I, actin binding/ actin filament/ cell-cell adhesion; II, apoptosis/ autophagy/ ubiquitination; III, growth factor; IV, immune response/ cytokine/ chemokine; V, inflammatory response; VI, metabolic process; VII, molecule transport/ ion transport; VIII, protein binding/ ion binding/ ATP binding/ chaperone; IX, signaling/ cell-cell communication; X, transcription; XI, uncharacterized. The number of genes belonging to a particular functional category is indicated between parentheses in each slice.
(TIF)

S4 Fig qPCR validation of DNA microarray data for STEC strains. In (A) are depicted the boxplots representing DNA microarray expression values for five selected genes in EH41 (circle) and in Ec472/01 (triangle) groups. In (B) are shown qPCR expression fold change boxplots for the same genes in EH41 or Ec472/01 groups.
(TIF)

S5 Fig qPCR validation DNA microarray data for Caco-2 cells. In (A) are depicted the boxplots representing DNA microarray expression values for five selected genes in Caco-2 cells interacting with EH41 (circle) or Ec472/01 (triangle) and control groups (square). In (B) are shown qPCR expression fold change boxplots for the same genes in those three groups.
(TIF)

S1 Table STEC strains used in this study. (DOCX)

S2 Table Primer sequences, product fragment length and PCR conditions used for gene detection by PCR in STEC strains. (DOCX)

S3 Table Primer sequences used for validation of gene expression by qPCR. (DOCX)

S4 Table Differentially expressed genes (DE and EE genes) obtained for EH41 strain after comparative global gene expression analysis of bacteria cultured in C x F medium. (DOCX)

S5 Table Differentially expressed genes (DE and EE genes) obtained for Ec472/01 strain after comparative global gene expression analysis of bacteria cultured in C x F medium. (DOCX)

S6 Table Differentially expressed genes (DE and EE genes) obtained for EH41 and for Ec472 after comparative global gene expression analysis of EH41 x Ec472/01 both cultured in C medium. (XLSX)
