# Supplementary figures and images for: A hemolytic-uremic syndrome-associated strain O113:H21 Shiga toxin-producing Escherichia coli specifically expresses a transcriptional module containing dicA and is related to gene network dysregulation in Caco-2 cells
Source: PLoS One. 2017 Dec 18;12(12):e0189613. doi: 10.1371/journal.pone.0189613 (PMC5734773; doi:10.1371/journal.pone.0189613)

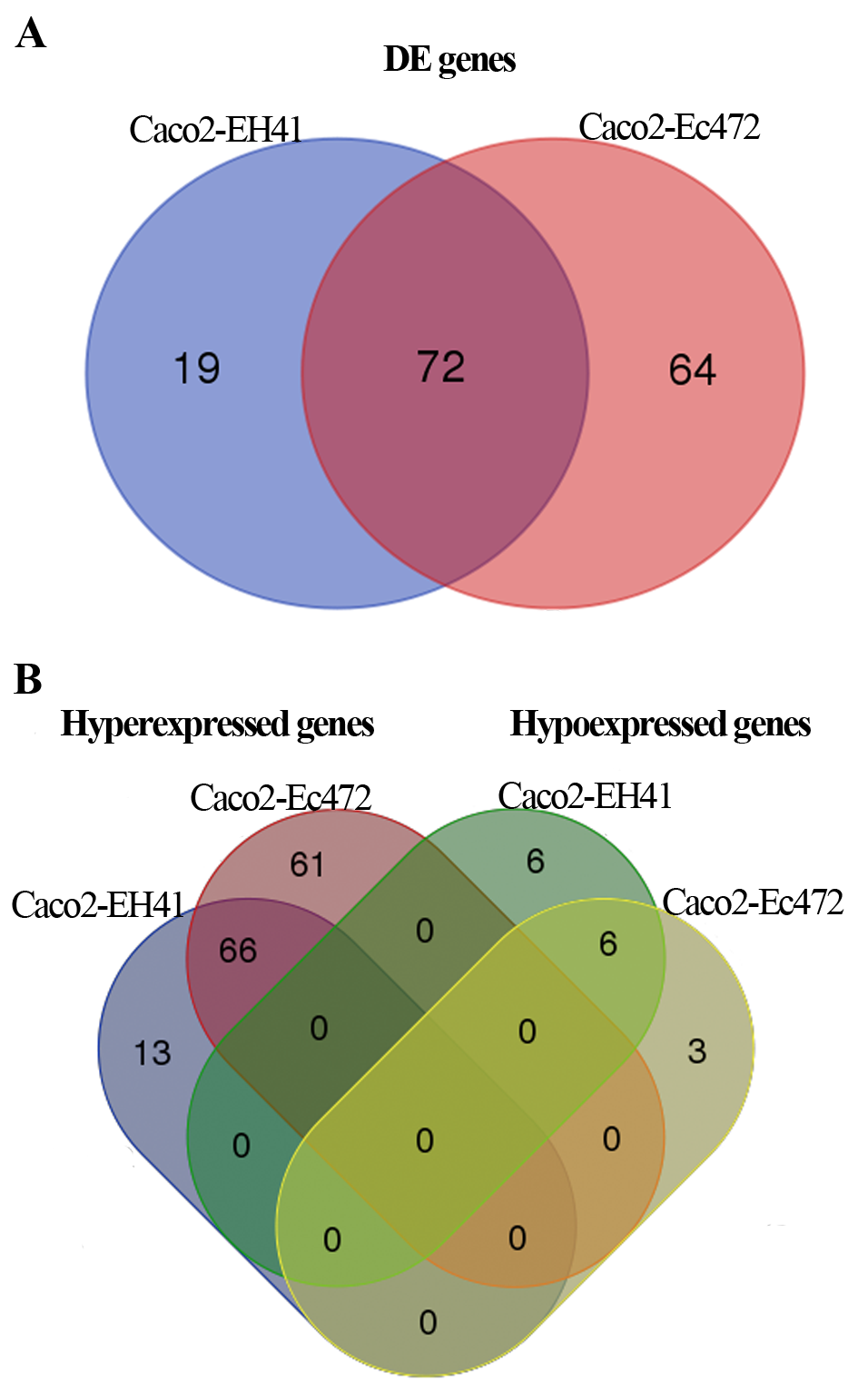

Supplement: S2 Fig — Venn diagram analysis of DE genes obtained from two comparisons: (A) Caco-2 with EH41X Caco-2 control or Caco-2 with Ec472/01 X Caco-2 control; (B) Venn diagram analysis of hyper and hypo expressed genes between the two groups. (TIF) [file pone.0189613.s002.tif]

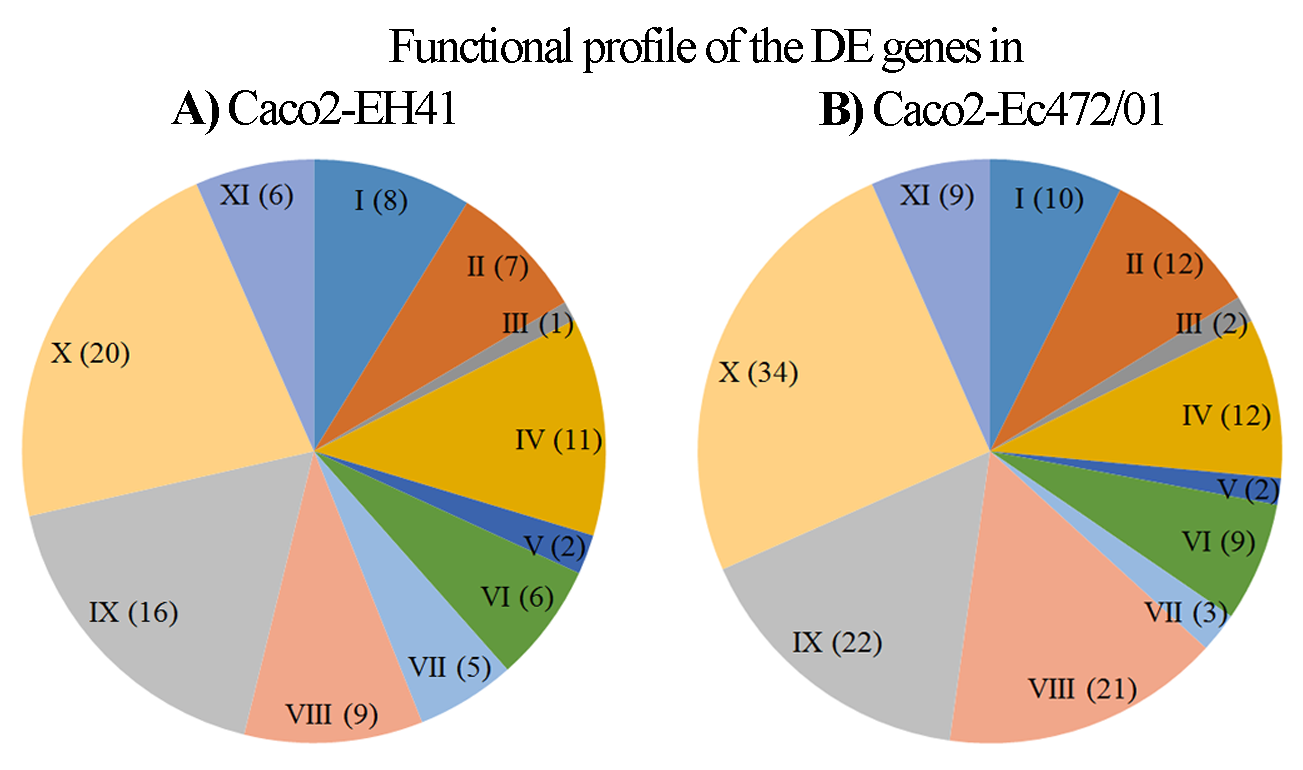

Supplement: S3 Fig — Pie charts of DE genes obtained from two comparative analyses: Caco-2 with EH41 X Caco-2 control (A) or Caco-2 with Ec472/01 X Caco-2 control (B). Functional categories are identified by roman numerals as follows: I, actin binding/ actin filament/ cell-cell adhesion; II, apoptosis/ autophagy/ ubiquitination; III, growth factor; IV, immune response/ cytokine/ chemokine; V, inflammatory response; VI, metabolic process; VII, molecule transport/ ion transport; VIII, protein binding/ ion binding/ ATP binding/ chaperone; IX, signaling/ cell-cell communication; X, transcription; XI, uncharacterized. The number of genes belonging to a particular functional category is indicated between parentheses in each slice. (TIF) [file pone.0189613.s003.tif]

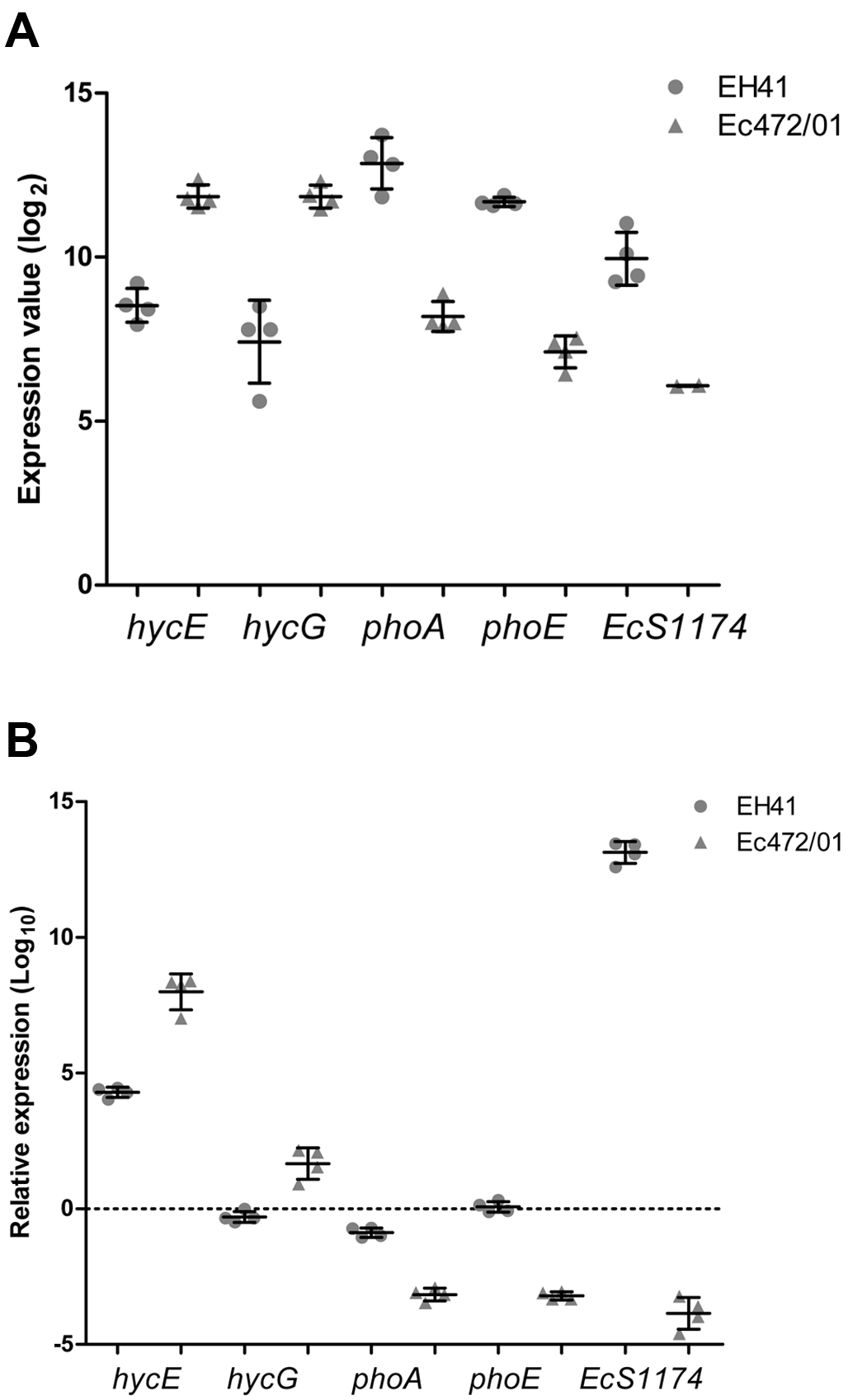

Supplement: S4 Fig — In (A) are depicted the boxplots representing DNA microarray expression values for five selected genes in EH41 (circle) and in Ec472/01 (triangle) groups. In (B) are shown qPCR expression fold change boxplots for the same genes in EH41 or Ec472/01 groups. (TIF) [file pone.0189613.s004.tif]

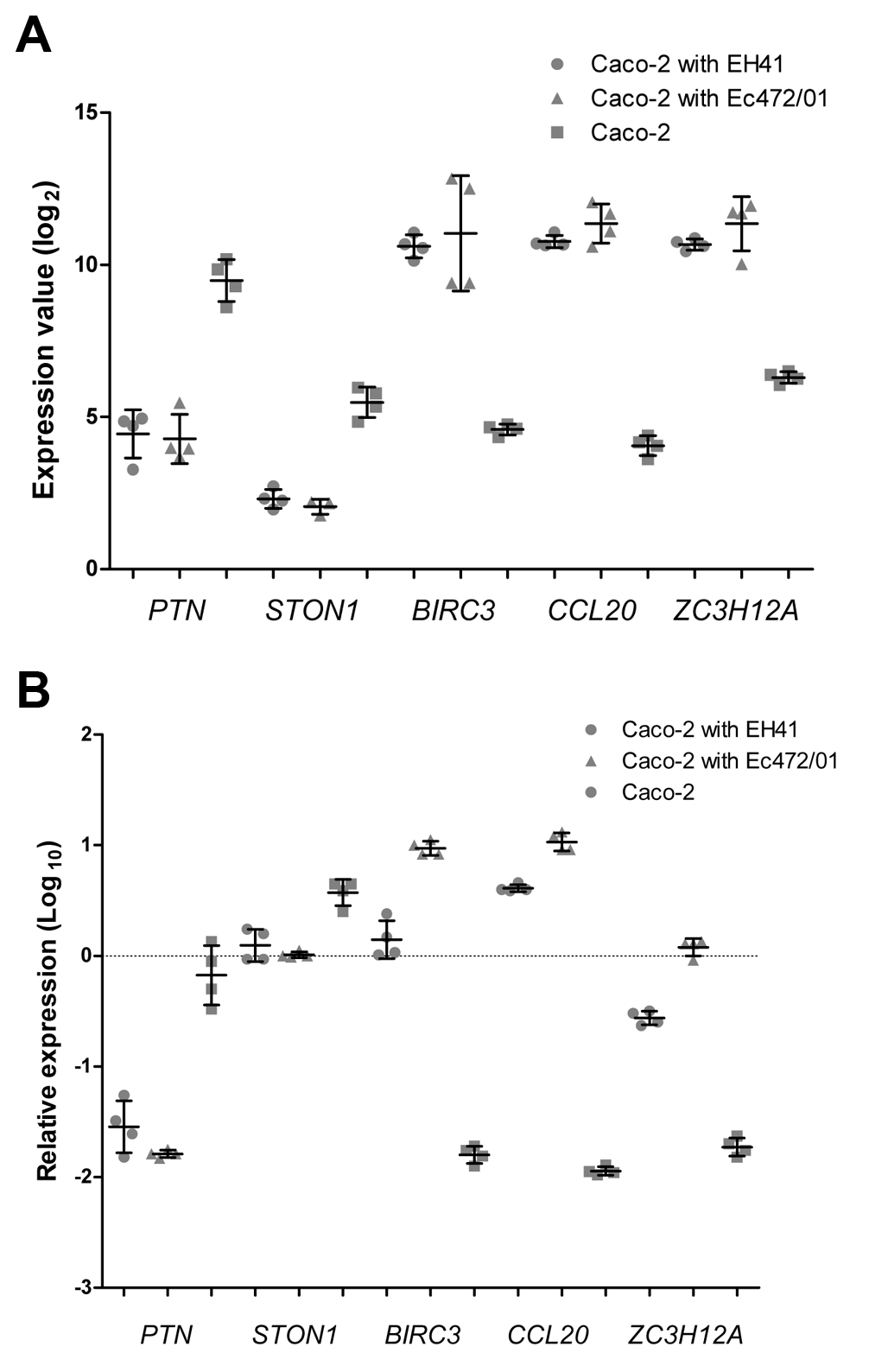

Supplement: S5 Fig — In (A) are depicted the boxplots representing DNA microarray expression values for five selected genes in Caco-2 cells interacting with EH41 (circle) or Ec472/01 (triangle) and control groups (square). In (B) are shown qPCR expression fold change boxplots for the same genes in those three groups. (TIF) [file pone.0189613.s005.tif]
