# Supplementary material for: A hemolytic-uremic syndrome-associated strain O113:H21 Shiga toxin-producing Escherichia coli specifically expresses a transcriptional module containing dicA and is related to gene network dysregulation in Caco-2 cells
Source: PLoS One. 2017 Dec 18;12(12):e0189613. doi: 10.1371/journal.pone.0189613 (PMC5734773; doi:10.1371/journal.pone.0189613)
Supplement: S1 Table — (DOCX) [file pone.0189613.s006.docx]

| **S1 Table**. STEC strains used in this study | |
| --- | --- |
| **Strain** | **Source** |
| EH41 | Patient with HUS |
| Ec472/01 | Bovine feces |
| 226/1 | Bovine feces |
| Ec670/05 | Bovine feces |
| Ec254/01 | Bovine feces |
| Ec226/04 | Bovine feces |
| Ec503/05 | Goat feces |
| Ec182/04 | Buffalo feces |
| Ec624/05 | Bovine feces |
| Ec684/04 | Bovine feces |
| Ec253/02 | Bovine feces |
| Ec784 | Beef meat |
