# Supplementary material for: A hemolytic-uremic syndrome-associated strain O113:H21 Shiga toxin-producing Escherichia coli specifically expresses a transcriptional module containing dicA and is related to gene network dysregulation in Caco-2 cells
Source: PLoS One. 2017 Dec 18;12(12):e0189613. doi: 10.1371/journal.pone.0189613 (PMC5734773; doi:10.1371/journal.pone.0189613)
Supplement: S2 Table — (DOCX) [file pone.0189613.s007.docx]

**S2 Table**. Primer sequences, product fragment length and PCR conditions used for gene detection by PCR in STEC strains

| **Gene** | **Primer foward (5'-3')** | **Primer reverse (5'-3')** | **Product lenght (bp)** | **MgSO4**  **(mM)** | **Temp**  **(°C)** |
| --- | --- | --- | --- | --- | --- |
| *ECs2098* | CAGACCTGGGACGACGAAAA | ATACCCTGCAGTTTCGGGTG | 1178 | 1.5 | 62 |
| *ECs1176* | CGCGGGCGATATTTTCACAG | GTACCGGATGTGTTCTGCCA | 285 | 2.0 | 60 |
| *dicA* | CGCATCAGGTATCGTCGGAA | TACTCGAGCTCGCATTTCGG | 315 | 2.0 | 60 |
| *dicC* | GCAGCAGGTATTCGTTTGGC | TTTCATTGTTCAACCGCCCC | 166 | 2.0 | 60 |
| *fecC* | AATGGCCTCTCCTTCACTGC | CATCCTCGCCTCCTCACAAG | 735 | 2.0 | 60 |
| *gadB* | CAGACCTGGGACGACGAAAA | ACTCATACGGCCCCAGTTTG | 901 | 2.0 | 60 |
| *insA* | CAGCGCTATCTCTGCTCTCC | CGTAAAACCGTGTTGAGGCC | 164 | 2.0 | 60 |
| *insI* | GTTAAGGGATACTGGCGGCA | CCAAGGACTCTGAGGATCGC | 844 | 1.0 | 57 |
| *rusA* | GCATCACATTACCCTGGCCT | CCCCATTTCGGTGATGGTCA | 341 | 2.0 | 60 |
