# Supplementary material for: A hemolytic-uremic syndrome-associated strain O113:H21 Shiga toxin-producing Escherichia coli specifically expresses a transcriptional module containing dicA and is related to gene network dysregulation in Caco-2 cells
Source: PLoS One. 2017 Dec 18;12(12):e0189613. doi: 10.1371/journal.pone.0189613 (PMC5734773; doi:10.1371/journal.pone.0189613)
Supplement: S3 Table — (DOCX) [file pone.0189613.s008.docx]

| **S3 Table**. Primer sequences used for validation of gene expression by qPCR | | | |  |
| --- | --- | --- | --- | --- |
| **Cell type** | **Gene** | **Primer foward (5'-3')** | **Primer reverse (5'-3')** | **Product lenght (bp)** |
| Caco-2 cells | *CCL20* | GCGCAAATCCAAAACAGACT | CAAGTCCAGTGAGGCACAAA | 280 |
|  | *ZC3H12A* | GCCGAGATCCTCTCCTACAA | CGACTTGAATCCTCCCTCTG | 350 |
|  | *PTN* | CTGCCTTCTTGGCATTCATT | TTCATGGTTTGCTTGCACTC | 490 |
|  | *STON1* | AGGAAAGGAATGAGGGAGGA | GCACAAATCATCTACCCATGAA | 490 |
|  | *BIRC3* | TGCAAGAAGCTGAAGCTGTG | GAAGGAGCACAATCTTTGCAT | 490 |
|  | *GAPDH* | ACCACAGTCCATGCCATCAC | TCCACCACCCTGTTGCTGTA | 451 |
| STEC | *hycE* | CCGTCTGAAAGTGCGTATCA | TGCTCCAGTGGATATCATCG | 264 |
|  | *phoE* | CCATCGCTGGGTTATGTCTT | TCGCTATCCAGTTGGTTGATT | 258 |
|  | *phoA* | TAGATAAGCCCGCAGTCACC | TATCGATTGACGCACCTTCA | 313 |
|  | *hycG* | AGCATGAAGGCGTCGTTACT | AACGACTTTAATGCCGAAGC | 271 |
|  | *Ecs1174* | ATGCCTGGTACTTTGCCAAC | AAACCAATTTCAGCCAGTGC | 408 |
|  | *rpoA* | GCGCTCATCTTCTTCCGAAT | CGCGGTCGTGGTTATGTG | 57 |
