# Supplementary material for: A hemolytic-uremic syndrome-associated strain O113:H21 Shiga toxin-producing Escherichia coli specifically expresses a transcriptional module containing dicA and is related to gene network dysregulation in Caco-2 cells
Source: PLoS One. 2017 Dec 18;12(12):e0189613. doi: 10.1371/journal.pone.0189613 (PMC5734773; doi:10.1371/journal.pone.0189613)
Supplement: S4 Table — (DOCX) [file pone.0189613.s009.docx]

| **S4 Table**. Differentially expressed genes (DE and EE genes) obtained for EH41 strain after comparative global gene expression analysis of bacteria cultured in C x F medium | | |
| --- | --- | --- |
|  | **Gene** | **Description** |
| **DE:**  **hypo-exp** | lamB | phage lambda receptor protein; maltose high-affinity receptor [b4036] |
|  | phnD | Phosphonates-binding periplasmic protein precursor [c_5110] |
|  | rffE | UDP-N-acetyl glucosamine -2-epimerase; synthesis of enterobacterial common antigen [b3786] |
|  | yedE | Hypothetical protein yedE [c_2344] |
| **DE:**  **hyper-exp** | c_0002 | Hypothetical protein [c_0002] |
|  | Z4176 | putative 2-component transcriptional regulator [Z4176] |
|  | alsC | putative transport system permease protein [b4086] |
| **EE** | ECs1620 | antitermination protein [ECs1620] |
|  | ECs3485 | chaperone-like protein [ECs3485] |
|  | topB | DNA topoisomerase III [c_2166] |
|  | emrE | EmrE protein; multidrug resistance protein [c_2352] |
|  | ECs4380 | heme utilization/transport protein [ECs4380] |
|  | ECs1070 | hypothetical protein [ECs1070] |
|  | ECs2274 | hypothetical protein [ECs2274] |
|  | ECs2758 | hypothetical protein [ECs2758] |
|  | ECs3499 | hypothetical protein [ECs3499] |
|  | ECs1201 | hypothetical protein NinG [ECs1201] |
|  | Z2241 | orf Unknown function [Z2241] |
|  | Z4400 | orf Unknown function [Z4400] |
|  | Z4888 | orf Unknown function [Z4888] |
|  | Z5117 | orf Unknown function [Z5117] |
|  | Z5894 | orf Unknown function [Z5894] |
|  | yafZ | orf, hypothetical protein [b0252] |
|  | yfbO | orf, hypothetical protein [b2274] |
|  | yfjZ | orf, hypothetical protein [b2645] |
|  | ECs4328 | putative acyl carrier protein [ECs4328] |
|  | Z4853 | putative acyl carrier protein [Z4853] |
|  | ECs0299 | putative DNA binding protein [ECs0299] |
|  | Z1965 | putative iron compound ABC transporter, permease protein [Z1965] |
|  | Z4383 | putative iron compound permease protein of ABC [Z4383] |
|  | ECs1374 | putative membrane protein [ECs1374] |
|  | ECs3221 | putative outer membrane protein [ECs3221] |
|  | psuI | putative polarity suppression protein encoded in CP-933I [Z0333] |
|  | Z1531 | putative regulator Not classified [Z1531] |
|  | ECs2989 | putative regulatory protein [ECs2989] |
|  | Z5614 | putative sorbose PTS component [Z5614] |
|  | Z1857 | unknown protein encoded by prophage CP-933C [Z1857] |
|  | Z2397 | unknown protein encoded within prophage CP-933R [Z2397] |
