# Supplementary material for: A hemolytic-uremic syndrome-associated strain O113:H21 Shiga toxin-producing Escherichia coli specifically expresses a transcriptional module containing dicA and is related to gene network dysregulation in Caco-2 cells
Source: PLoS One. 2017 Dec 18;12(12):e0189613. doi: 10.1371/journal.pone.0189613 (PMC5734773; doi:10.1371/journal.pone.0189613)
Supplement: S5 Table — (DOCX) [file pone.0189613.s010.docx]

| **S5 Table**. Differentially expressed genes (DE and EE genes) obtained for Ec472/01 strain after comparative global gene expression analysis of bacteria cultured in C x F medium | | |
| --- | --- | --- |
|  | **Gene** | **Description** |
| **DE:**  **hypo-exp** | *entA* | 2,3-dihydro-2,3-dihydroxybenzoate dehydrogenase, enterochelin biosynthesis [b0596] |
|  | *entF* | Enterobactin synthetase component F [c_0673] |
|  | *cspG* | homolog of Salmonella cold shock protein [b0990] |
| **DE:**  **hyper-exp** | *metF* | 5,10-methylenetetrahydrofolate reductase [b3941] |
|  | *adiA* | biodegradative arginine decarboxylase [b4117] |
|  | *cbpA* | curved DNA-binding protein; functions closely related to DnaJ [b1000] |
|  | *cfa* | cyclopropane fatty acyl phospholipid synthase [b1661] |
|  | *dadA* | D-amino acid dehydrogenase small subunit [c_1638] |
|  | *lfhA* | Pseudogene; flagellar biosynthesis [b0229] |
|  | *dps* | global regulator, starvation conditions [b0812] |
|  | *hyaB* | hydrogenase-1 large subunit [b0973] |
|  | *cadA* | lysine decarboxylase 1 [b4131] |
|  | *trg* | methyl-accepting chemotaxis protein III, ribose sensor receptor [b1421] |
|  | *narI* | nitrate reductase 1, cytochrome b [b1227] |
|  | *narJ* | nitrate reductase 1, delta subunit, assembly function [b1226] |
|  | *narK* | nitrite extrusion protein [b1223] |
|  | *appA* | Periplasmic appA protein precursor [c_1121] |
|  | *hyaD* | processing of HyaA and HyaB proteins [b0975] |
|  | *metR* | regulator for metE and metH [Z5349] |
|  | *cusS* | Sensor kinase cusS [c_0656] |
|  | *sodB* | Superoxide dismutase [c_2050] |
|  | *ECs4759* | tetrahydropteroyltriglutamate methyltransferase [ECs4759] |
|  | *ECs0037* | transcriptional regulator of cai operon [ECs0037] |
|  | *cadB* | transport of lysine/cadaverine [b4132] |
|  | *wrbA* | trp repressor binding protein; affects association of trp repressor and operator [b1004] |
|  | *ECs1889* | hypothetical protein [ECs1889] |
|  | *ECs2902* | hypothetical protein [ECs2902] |
|  | *ECs2955* | hypothetical protein [ECs2955] |
|  | *yeeA* | Hypothetical protein yeeA [c_2536] |
|  | *yjfY* | Hypothetical protein yjfY precursor [c_5289] |
|  | *yohC* | Hypothetical protein yohC [c_2667] |
|  | *yihW* | Hypothetical transcriptional regulator yihW [c_4824] |
|  | *cbpM* | orf, hypothetical protein [b0999] |
|  | *yccJ* | orf, hypothetical protein [b1003] |
|  | *ydfZ* | orf, hypothetical protein [b1541] |
|  | *yohN* | orf, hypothetical protein [b2107] |
|  | *yjjU* | orf, hypothetical protein [b4377] |
|  | *hyaC* | Probable Ni/Fe-hydrogenase 1 B-type cytochrome subunit [c_1115] |
|  | *appB* | probable third cytochrome oxidase, subunit II [b0979] |
|  | *ygbE* | putative cytochrome oxidase subunit [b2749] |
|  | *ECs1542* | putative large terminase subunit [ECs1542] |
|  | *yohK* | putative seritonin transporter [b2142] |
|  | *yfcG* | putative S-transferase [b2302] |
|  | *c_1579* | Putative tail component of prophage [c_1579] |
|  | *yeaU* | putative tartrate dehydrogenase [b1800] |
| **EE** | *rplP* | 50S ribosomal subunit protein L16 [b3313] |
|  | *lnt* | apolipoprotein N-acyltransferase, copper homeostasis protein, inner membrane [b0657] |
|  | *fdhD* | FdhD protein [c_4847] |
|  | *hyfC* | hydrogenase 4 membrane subunit [b2483] |
|  | *ECs1070* | hypothetical protein [ECs1070] |
|  | *ECs4328* | putative acyl carrier protein [ECs4328] |
|  | *yddL* | putative outer membrane porin protein [b1472] |
|  | *ybhD* | putative transcriptional regulator LYSR-type [b0768] |
